# FTO controls CD8^+^ T cell survival and effector response by modulating m^6^A methylation of Fas

**DOI:** 10.1038/s41419-025-07606-z

**Published:** 2025-04-15

**Authors:** Lina Sun, Tianzhe Zhang, Yao Ge, Zhihong Yao, Yanhong Su, Qianhao Wang, Yang Chen, Boxiao He, Renyi Ding, Cangang Zhang, Linbo Lan, Ruonan Liu, Huanxin Ping, Dan Zhang, Lin Shi, Xiaobo Zhou, Xiaoxuan Jia, Chenming Sun, Lingli Liang, Lianjun Zhang, Baojun Zhang

**Affiliations:** 1 Department of Pathogenic Microbiology and Immunology, School of Basic Medical Sciences, Xi’an Jiaotong University, Xi’an, Shaanxi China; 2 Institute of Infection and Immunity, Translational Medicine Institute, Xi’an Jiaotong University Health Science Center, Xi’an, Shaanxi China; 3 Key Laboratory of Environment and Genes Related to Diseases, Ministry of Education, Xi’an, Shaanxi China; 4 Xi’an Key Laboratory of Immune Related Diseases, Xi’an, Shannxi China; 5 Department of Human Anatomy and Histoembryology, School of Basic Medical Sciences, Xi’an Jiaotong University, Xi’an, Shaanxi China; 6 Faculty of Clinical Medicine, Hanzhong Vocational and Technical College, Hanzhong, China; 7 Clinical Teaching and Research Center, School of Nursing, Weinan Vocational and Technical College, Weinan, Shaanxi China; 8 Department of Physiology and Pathophysiology, School of Basic Medical Sciences, Xi’an Jiaotong University Health Science Center, Xi’an, Shaanxi, 710061, PR China; Institute of Neuroscience, Translational Medicine Institute, Xi’an Jiaotong University Health Science Center, Xi’an, Shaanxi PR China; 9 National Key Laboratory of Immunity and Inflammation, Suzhou Institute of Systems Medicine, Chinese Academy of Medical Sciences & Peking Union Medical College, Suzhou, Jiangsu China; 10 Key Laboratory of Synthetic Biology Regulatory Elements, Suzhou Institute of Systems Medicine, Chinese Academy of Medical Sciences & Peking Union Medical College, Suzhou, 215123 Jiangsu China

**Keywords:** Cell death and immune response, CD8-positive T cells, Epigenetics in immune cells

## Abstract

Functional CD8^+^ T cell immunity is essential for immune surveillance and host defense against infection and tumors. Epigenetic mechanisms, particularly RNA modification, in controlling CD8^+^ T cell immune response is not fully elucidated. Here, by T cell-specific deletion of fat mass and obesity-associated protein (FTO), a critical N6-methyladenosine (m^6^A) demethylase, we revealed that FTO was indispensable for adequate CD8^+^ T cell immune response and protective function. FTO ablation led to considerable cell death in activated CD8^+^ T cells, which was attributed to cell apoptosis. MeRIP-seq analysis revealed an increase in m^6^A methylation on *Fas* mRNA in FTO-deficient CD8^+^ T cells. The loss of FTO promoted Fas expression via enhancing the *Fas* mRNA stability, which depended on the m^6^A reader insulin-like growth factor-2 mRNA-biding proteins 3 (IGF2BP3). Mutation of the *Fas* m^6^A sites or knockdown IGF2BP3 could normalize the upregulated Fas expression and apoptosis levels caused by FTO ablation in CD8^+^ T cells. Our findings delineate a novel epigenetic regulatory mechanism of FTO-mediated m^6^A modification in supporting CD8^+^ T cell survival and effector responses, providing new insights into understanding the post-transcriptional regulation in CD8^+^ T cell immunological functions and the potential therapeutic intervention.

## Introduction

CD8^+^ T cells are pivotal cell populations in adaptive immunity for protecting the host against pathogenic infections and eliminating malignancy [1]. Upon recognizing the antigen, naïve CD8^+^ T cells undergo rapid activation, expansion, and differentiation into effector cells, which carry out cytotoxic function and memory cells afterwards, which provide long-term protection [2]. In response to acute infections, antigen-specific CD8^+^ T cells experience distinct stages: expansion, contraction (with massive apoptosis), and memory differentiation [3]. The CD8^+^ T cell immune responses are orchestrated by complex signal cascades, including T cell receptor (TCR), co-stimulation, cytokines, and transcription factors (TFs) [2]. In addition, epigenetic regulations, such as DNA methylation and histone modifications, have gained much attention in modulating CD8^+^ T cell differentiation and function by altering the chromatin accessibility of the regulatory regions within key genes in CD8^+^ T cells [4, 5]. Besides DNA and protein, RNA-based epigenetic regulations, including small RNAs, long non-coding RNAs (lncRNAs), and RNA modifications, have recently emerged as crucial regulators of gene expression, silencing, and chromatin structure, involving various biological processes [6–8]. The regulatory mechanisms mediated by small RNAs and lncRNAs have been reported to govern CD8^+^ T cell effector functions [9]. However, the contributions of post-transcriptional RNA modifications to CD8^+^ T cell immune response remain largely unknown. m^6^A is the most abundant and prevalent RNA (particularly mRNA) modification in eukaryotic cells. m^6^A modification is dynamically and reversibly modulated by methyltransferases (writers) consisting of methyltransferase 3 (METTL3), METTL14, Wilms Tumor Associated Protein (WTAP), vir-like m^6^A methyltransferase associated (VIRMA) etc., and demethylases (erasers) including two members, alpha-ketoglutarate-dependent dioxygenase ABH5 (ALKBH5) and FTO [10]. m^6^A modifications affect mRNAs' fate and downstream functions, such as decay, splicing, subcellular translocation, and translation, which are determined by m^6^A binding proteins (readers) [11]. To date, three distinct groups of proteins have been identified as m^6^A readers: YT521-B homology (YTH)-RNA binding domain family, including YTH domain family 1–3 (YTHDF1-3) and YTH domain containing 1–2 (YTHDC1-2); the heterogeneous nuclear ribonucleoprotein (HNRNP) family members (e.g. HNRNPC, HNRNPG, and HNRNPA2B1); and insulin-like growth factor-2 mRNA-biding proteins 1–3 (IGF2BP1-3) [12]. Among those readers, YTHDF2 has been shown to mediate mRNA decay, and YTHDF1, YTHDF3, and YTHDC2 promote translation, while IGF2BPs mainly enhance mRNA stability [13]. Previous studies have demonstrated that m^6^A modifications exert pivotal regulatory effects on T cell homeostasis and functionality [14]. For instance, m^6^A writer METTL3 was required for T cells homeostasis by regulating the IL-7/STAT5/SOCS pathway [15]. In addition, m^6^A modifications directly modulated T cell differentiation as METTL3 ablation in T cells exhibited disrupted T helper (Th)1 [15], Th17 [15], regulatory T (Treg) [16] and T follicular helper T (Tfh) [17] cell differentiation, as well as impaired CD8^+^ T cell effector differentiation and memory formation [18]. Moreover, m^6^A modifications can influence T cell immune response indirectly by affecting antigen-presenting cells (APCs), such as dendritic cells (DCs) [19] and macrophages [20, 21].

FTO is the first identified m^6^A demethylase and primarily functions to remove m^6^A methylation through its oxidative demethylase activity, playing critical roles in a variety of pathological conditions, including obesity, diabetes, heart failure, neurological disorders, and tumorigenesis [22–24]. However, the roles of FTO in modulating immune cell functions have not been well understood, with limited studies focusing on innate immune cells. FTO promotes polarization of both M1 and M2 macrophages by sustaining NF-κB signaling pathway and the mRNA stability of *STAT1* and *PPAR-γ* [25]. However, under diabetic conditions, FTO inhibits M1 macrophages activation and inflammation via m^6^A-regulated distinct signaling pathways [26, 27]. Additionally, FTO negatively regulates the cytotoxic activity of natural killer (NK) cells by increasing the mRNA stability of suppressor of cytokine signaling protein (SOCS) family genes [28]. While the direct regulatory roles of FTO in T cells remain unclear, indirect effects have been reported in the context of tumors, where FTO influences T cell function by modulating tumor cells [29, 30]. For example, FTO supports cancer (stem) cell growth and maintenance and the expression of immune checkpoint molecule PD-L1, thereby enhancing immune evasion [31–33]. Recently, FTO-mediated m^6^A demethylation promotes glycolytic activity in tumor cells, which impedes CD8^+^ T cell infiltration and effector function [24, 34]. Here, by T cell-specific deletion of FTO, we unveiled that FTO was crucial for supporting CD8^+^ T cell immune response and protective function during pathogenic infection. Ablation of FTO resulted in a pronounced increase in CD8^+^ T cell apoptosis upon activation, which was attributed to the upregulated expression of Fas receptor. Mechanistically, FTO deficiency caused an elevated level of m^6^A methylation on *Fas* mRNA, which enhanced the mRNA stability and, thereby, Fas expression in a manner dependent on the m^6^A reader protein IGF2BP3. Our findings define a crucial role of RNA epigenetic regulator via FTO-mediated m^6^A modification in CD8^+^ T cell survival and effector response.

## Results

### FTO is required for effector CD8^+^ T cell responses during acute infection

**Fig. 1:**
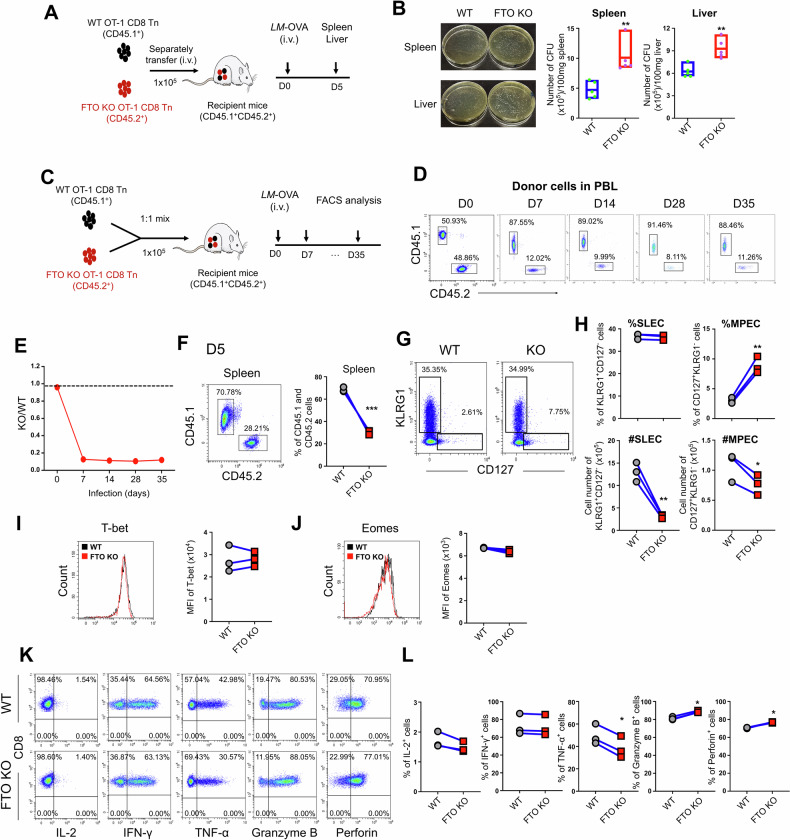
FTO is required for effector CD8^+^ T cell responses during acute infection. **A** Experimental diagram of in vivo CD8^+^ T cell separate transfer assay to assess the bacterial clearance. Five days after infection, the bacterial loads were determined in the spleen and liver from mice received WT or FTO KO CD8^+^ T cells (*n* = 5). **B** Representative photos of agar plates showing the bacterial colony formation in the spleen and liver; numbers of bacterial colonies per 100 mg tissues. **C** Experimental schematic of CD8^+^ T cell co-transfer model. **D** Flow cytometry analysis of donor cells from WT (CD45.1^+^) or FTO KO (CD45.2^+^) groups in CD8^+^ T cells in peripheral blood lymphocytes (PBL) at different timepoints after *LM*-OVA infection (*n* = 7). **E** The ratio of percentages of KO to WT cells in the PBL at different timepoints. **F**–**L** The proportions and phenotypes of donor-derived (OT-1^+^) CD8^+^ T cells from WT and KO groups were analyzed 5 days after *LM*-OVA infection in the spleen (*n* = 3). **F** Flow cytometry analysis of donor-derived (OT-1^+^) CD8^+^ T cells from WT (CD45.1^+^) and KO (CD45.2^+^) groups. **G** Representative flow cytometry plots of KLRG1 and CD127 expression in donor-derived (OT-1^+^) CD8^+^ T cells. **H** The proportion (%) and cell number (#) of KLRG1^+^CD127^−^ SLECs and KLRG1^−^CD127^+^ MPECs in donor-derived CD8^+^ T cells. Flow cytometry analysis of T-bet (**I**) and Eomes (**J**) expression in WT and FTO KO donor-derived (OT-1^+^) CD8^+^ T cells on day 5. Representative flow cytometry plots (**K**) and percentages (**L**) of WT and KO CD8^+^ T cells producing cytokines IL-2, IFN-γ, TNF-α, granzyme B, and perforin among donor-derived (OT-1^+^) cells on day 5. Data are representative of two or three independent experiments shown as the mean ± SD. Statistical testing is depicted as two-sided, unpaired *t*-tests; *P ≤ 0.05, **P ≤ 0.01, ***P ≤ 0.001.

To explore the effects of FTO-mediated m^6^A modification on CD8^+^ T cell function, we first generated T cell-specific *Fto* knockout (KO) mice (*Fto*^fl/fl^CD4*-*Cre^*+*^) and (*Fto*^fl/fl^CD4*-*Cre^*+*^OT-1^+^) mice by crossing *Fto*^fl/fl^ mice with CD4*-*Cre transgenic strain, and further crossing with OT-1 transgenic mice in which the T cells express TCR specific for ovalbumin peptide (OVA_257-264_) SIINFEKL (Supplementary Fig. 1A, B). The deletion of FTO was confirmed by quantitative PCR (qPCR) at the mRNA level in CD8^+^ T cells from FTO KO (*Fto*^fl/fl^CD4*-*Cre^*+*^) mice compared to wild-type (WT) (*Fto*^fl/fl^CD4*-*Cre^−^) mice (Supplementary Fig. 1C). FTO ablation in T cells did not affect T cell development under the homeostatic conditions (Supplementary Fig. 2), which was consistent with the previous study [35]. However, FTO is essential for the protective function of CD8^+^ T cells against pathogenic infections. In an in vivo acute infection model (Fig. 1A), naïve CD8^+^ T cells from WT (*Fto*^fl/fl^CD4-Cre^−^OT-1^+^, CD45.1^+^) and FTO KO (*Fto*^fl/fl^CD4-Cre^+^OT-1^+^, CD45.2^+^) mice were transferred separately into the WT recipient mice (CD45.1^+^CD45.2^+^) followed by infection with *Listeria monocytogenes* (*LM*) expressing OVA (*LM*-OVA). Bacterial load was determined in the spleen and liver tissues 5 days post-infection. The results showed that mice transferred with FTO KO CD8^+^ T cells, compared to those with WT CD8^+^ T cells, had significantly increased bacterial colonies on agar plates in both spleen and liver (Fig. 1B), suggesting that FTO-deficient CD8^+^ T cells had impaired effector function against infection.

To further determine the intrinsic role of FTO in CD8^+^ T cells, we next co-transferred naïve CD8^+^ T cells from WT (*Fto*^fl/fl^CD4-Cre^−^OT-1^+^, CD45.1^+^) and FTO KO (*Fto*^fl/fl^CD4-Cre^+^OT-1^+^, CD45.2^+^) mice at a ratio of 1:1 into the same WT recipient mice (CD45.1^+^CD45.2^+^). After *LM*-OVA infection, the CD8^+^ T cell immune response in the secondary lymphoid tissue was monitored at different time periods (Fig. 1C). Compared to an equal number transferred on day 0, the ratio of FTO KO to WT OT-1^+^CD8^+^ T cells was significantly reduced in peripheral blood lymphocytes (PBL) following infection (Fig. 1D, E), strongly suggesting that FTO-deficient CD8^+^ T cells had a diminished immune response. Given that CD8^+^ T cell expansion reaches its peak around day 7 [2], we further examined the CD8^+^ T cell response at earlier timepoints before the expansion peak in the acute infection model. Consistently, FTO KO OT-1^+^CD8^+^ T cells exhibited a remarkable reduction in cell expansion on day 4, 5, and 6 in the spleen compared to WT counterparts (Fig. 1F and Supplementary Fig. 3A, B). Based on the expression of KLRG1 and CD127, effector CD8^+^ T cells at the peak of expansion can be divided into two subpopulations: KLRG1^+^CD127^−^ terminally differentiated short-lived effector cells (SLECs) and KLRG1^−^CD127^+^ long-lived memory precursor effector cells (MPECs) [36]. The evaluation of CD8^+^ T cell differentiation revealed that the frequencies of SLECs were comparable between WT and FTO KO groups, while the frequencies of MPECs were increased on days 4 and 5 but unchanged on day 6 (Fig. 1G, H and Supplementary Fig. 3C–F). However, owing to the massive reduction of total FTO KO CD8^+^ T cells, the absolute cell numbers of both SLECs and MPECs were decreased during the immune response (Fig. 1G, H and Supplementary Fig. 3C–F). Consistently, there was no difference in the expression of effector and memory CD8^+^ T cell-specific TFs T-bet and Eomes between WT and FTO KO CD8^+^ T cells (Fig. 1I, J). In addition, FTO-deficient CD8^+^ T cells displayed comparable cytokine production of IL-2 and IFN-γ and very slightly changed TNF-α, granzyme B, and perforin compared to their WT counterparts (Fig. 1K, L). Similarly, no differences in cytokine production were also observed in WT (*Fto*^fl/fl^CD4-Cre^−^OT-1^+^) and FTO KO (*Fto*^fl/fl^CD4-Cre^+^OT-1^+^) CD8^+^ T cells stimulated in vitro with OVA_257-264_ peptide (Supplementary Fig. 3G). Collectively, these data elucidate a pivotal role of FTO in facilitating the CD8^+^ T cell population expansion during acute response, yet with limited effects on effector/memory cell differentiation and cytokine production.

### FTO deficiency affects CD8^+^ T cell survival during immune responses

**Fig. 2:**
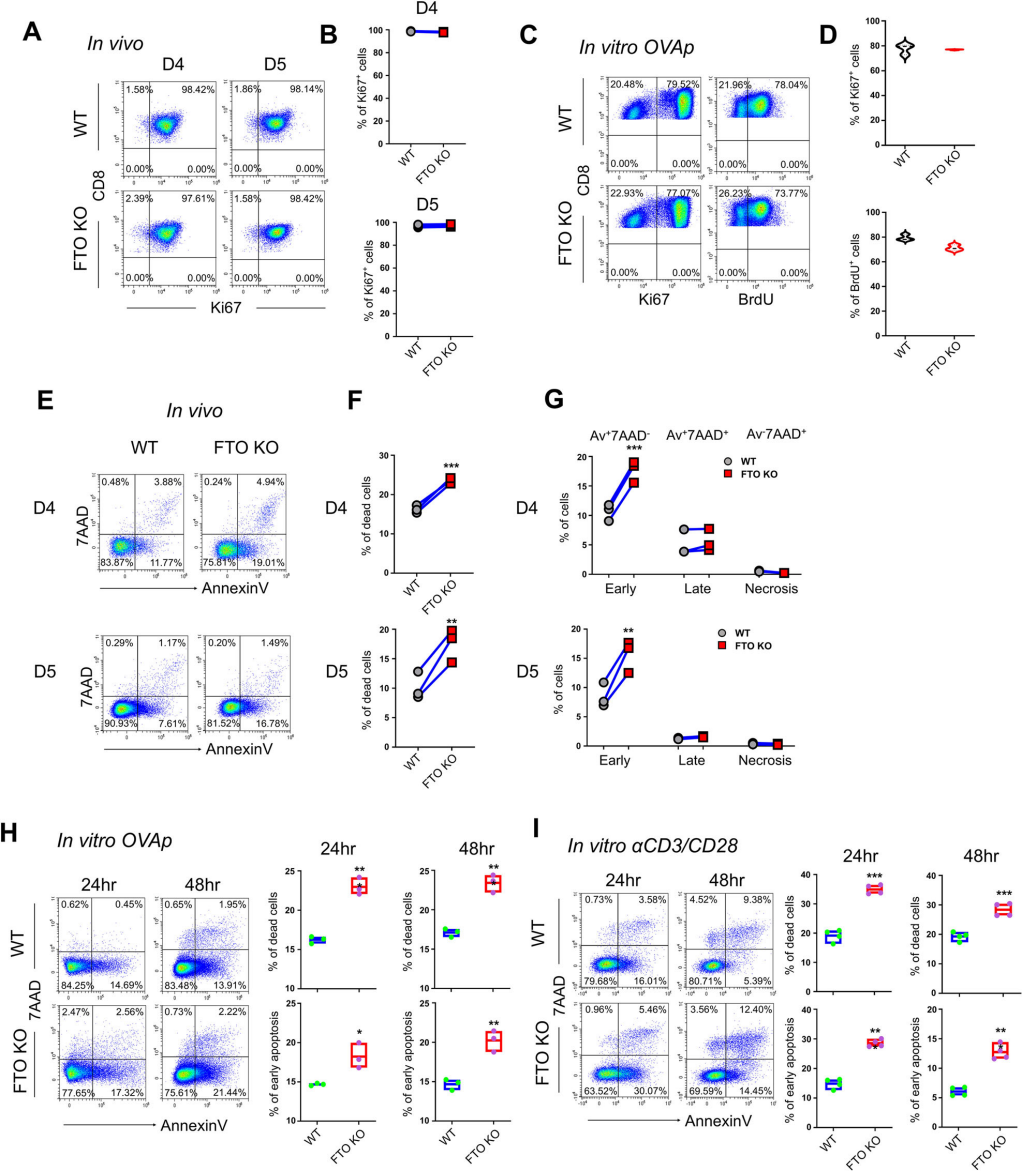
FTO deficiency affects effector CD8^+^ T cell survival. **A**, **B** Flow cytometry analysis of Ki67 expression in WT and FTO KO donor-derived (OT-1^+^) CD8^+^ T cells in the spleen from recipient mice 4 (left) and 5 (right) days post *LM*-OVA infection in the in vivo co-transfer model in Fig. 1C (*n* = 3). **C**, **D** Flow cytometry analysis of Ki67 expression and BrdU incorporation in WT (*Fto*^fl/fl^CD4-Cre^−^OT-1^+^) and FTO KO (*Fto*^fl/fl^CD4-Cre^+^OT-1^+^) CD8^+^ T cells stimulated in vitro with OVA_257-264_ peptide for 48 h (*n* = 3). **E** Flow cytometry analysis of Annexin V and 7AAD expression in WT and FTO KO donor-derived (OT-1^+^) CD8^+^ T cells in the spleen from recipient mice 4 (up) or 5 (bottom) days post *LM*-OVA infection in the in vivo co-transfer model (*n* = 3). **F** The frequencies of total dead (Annexin V^+^ and 7AAD^+^) cells in WT and FTO KO donor-derived (OT-1^+^) CD8^+^ T cells 4 (up) or 5 (bottom) days post-infection. **G** The frequencies of early (Annexin V^+^7AAD^−^) and late (Annexin V^+^7AAD^+^) apoptotic cells and necroptotic (Annexin V^−^7AAD^+^) cells in WT and FTO KO donor-derived (OT-1^+^) CD8^+^ T cells 4 (up) or 5 (bottom) days post-infection. **H**, **I** Representative flow cytometry plots and the frequencies of Annexin V and 7AAD expression in WT and FTO KO CD8^+^ T cells upon stimulation. **H** Splenocytes from WT (*Fto*^fl/fl^CD4-Cre^−^OT-1^+^) and FTO KO (*Fto*^fl/fl^CD4-Cre^+^OT-1^+^) mice were isolated and stimulated in vitro with OVA_257-264_ peptide for 24 h or 48 h to measure Annexin V and 7AAD expression in CD8^+^ T cells (*n* = 3). **I** Splenocytes from WT (*Fto*^fl/fl^CD4-Cre^−^) and FTO KO (*Fto*^fl/fl^CD4-Cre^+^) mice were isolated and stimulated in vitro with anti-CD3/CD28 antibodies for 24 h or 48 h to measure Annexin V and 7AAD expression in CD8^+^ T cells (*n* = 4). Percentages of total dead and early apoptotic cells were shown in (**H**, **I**). Data are representative of two or three independent experiments shown as the mean ± SD. Statistical testing is depicted as two-sided, unpaired *t*-tests; *P ≤ 0.05, **P ≤ 0.01, ***P ≤ 0.001.

Upon antigen stimulation, CD8^+^ T cells undergo rapid cell activation, proliferation, and apoptosis before developing into effector and memory T cells [2]. To further explore the underlying mechanisms responsible for the diminished CD8^+^ T cell population after FTO ablation, we assessed the cellular activation, proliferation, and cell death in WT and FTO-deficient CD8^+^ T cells in both the in vitro stimulation model and in vivo co-transfer model. When naïve CD8^+^ T cells from WT (*Fto*^fl/fl^CD4*-*Cre^−^OT-1^+^) and FTO KO (*Fto*^fl/fl^CD4*-*Cre^*+*^OT-1^+^) mice were stimulated in vitro with cognate peptide (OVA_257-264_), FTO-deficient OT-1^+^CD8^+^ T cells, compared to WT controls, showed comparable T cell activation after 24 h assessed by the expression of activation markers CD69 and CD25 (Supplementary Fig. 4A, B). Similarly, when naïve CD8^+^ T cells from WT (*Fto*^fl/fl^CD4*-*Cre^−^) and FTO KO (*Fto*^fl/fl^CD4*-*Cre^*+*^) mice were stimulated in vitro with anti-CD3/CD28 antibodies, T cell activation after 24 h was also unaltered between the two groups (Supplementary Fig. 4C, D). In terms of cell proliferation, we first measured the expression of cell-cycle marker Ki67 on CD8^+^ T cells obtained from the in vivo co-transfer model (Fig. 1C) at the expansion phase [2]. The results showed that FTO ablation did not affect CD8^+^ T cell proliferation on days 4 and 5, as evidenced by comparable expression of Ki67 (Fig. 2A, B). Similar results were observed with BrdU incorporation in WT and KO CD8^+^ T cells in the in vivo co-transfer model (Supplementary Fig. 4E). Moreover, when stimulated in vitro with OVA_257-264_ peptide, FTO-deficient OT-1^+^CD8^+^ T cells also exhibited unchanged proliferation determined by both Ki67 staining and BrdU incorporation (Fig. 2C, D).

Nevertheless, FTO deficiency in CD8^+^ T cells caused massive cell death upon antigen stimulation. In the in vivo co-transfer model, FTO KO CD8^+^ T cells had significantly increased expression levels of Annexin V and 7AAD on both day 4 and 5 post-infection (Fig. 2E, F). Specifically, the proportions of Annexin V^+^7AAD^−^ early apoptotic cells, but not Annexin V^+^7AAD^+^ late apoptotic cells and Annexin V^−^7AAD^+^ necroptotic cells, were markedly elevated in FTO KO CD8^+^ T cells (Fig. 2G). Consistently, the frequencies of total apoptotic and early apoptotic cells were significantly increased in FTO-deficient CD8^+^ T cells stimulated in vitro with OVA_257-264_ peptide (OT-1^+^CD8^+^ T cells) (Fig. 2H) and anti-CD3/CD28 antibodies (WT CD8^+^ T cells) (Fig. 2I) for both 24 h and 48 h. These data indicate that FTO deletion has a limited impact on CD8^+^ T cell activation and proliferation but causes significant cell death during immune responses.

### FTO deficiency results in increased CD8^+^ T cell apoptosis

**Fig. 3:**
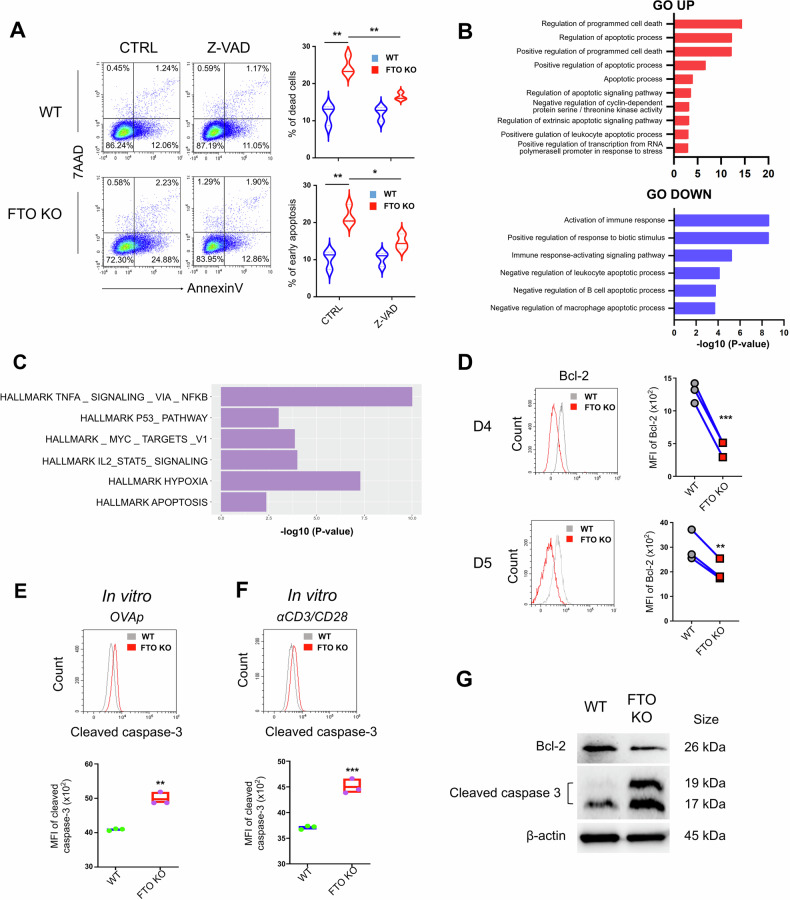
FTO-deficient effector CD8^+^ T cells have increased cell apoptosis. **A** Splenocytes from WT (*Fto*^fl/fl^CD4-Cre^−^) and FTO KO (*Fto*^fl/fl^CD4-Cre^+^) mice were stimulated with anti-CD3/CD28 antibodies for 24 h in the presence of Z-VAD to measure Annexin V and 7AAD expression in CD8^+^ T cells (*n* = 3). **B** GO analysis of DEGs from RNA-seq data depicting the upregulated and downregulated signaling pathways in FTO KO CD8^+^ T cells compared to WT control. **C** Hallmark gene sets associated signaling pathways were enriched in FTO KO CD8^+^ T cells. **D** Representative flow cytometry plots and the mean fluorescence intensity (MFI) of Bcl-2 expression in WT and FTO KO donor-derived (OT-1^+^) CD8^+^ T cells in the spleen from recipient mice 4 (up) or 5 (bottom) days post *LM*-OVA infection in the in vivo co-transfer model (*n* = 3). **E** Representative flow cytometry plots and the MFI of activated (cleaved) caspase-3 expression in the WT (*Fto*^fl/fl^CD4-Cre^−^OT-1^+^) and FTO KO (*Fto*^fl/fl^CD4-Cre^+^OT-1^+^) CD8^+^ T cells stimulated in vitro with OVA_257-264_ peptide (left) for 24 h (*n* = 3). **F** Representative flow cytometry plots and the MFI of activated (cleaved) caspase-3 expression in the WT (*Fto*^fl/fl^CD4-Cre^−^) and FTO KO (*Fto*^fl/fl^CD4-Cre^+^) CD8^+^ T cells stimulated in vitro with anti-CD3/CD28 antibodies for 24 h (*n* = 3). **G** The protein levels of Bcl-2 and cleaved caspase-3 were measured by Western blot in WT (*Fto*^fl/fl^CD4-Cre^−^) and FTO KO (*Fto*^fl/fl^CD4-Cre^+^) CD8^+^ T cells stimulated in vitro with anti-CD3/CD28 antibodies for 48 h. Data are representative of two or three independent experiments shown as the mean ± SD. Statistical testing is depicted as two-sided, unpaired *t*-tests or two-way ANOVA; *P ≤ 0.05, **P ≤ 0.01, ***P ≤ 0.001.

To reveal the mechanisms underlying the T cell death in FTO KO CD8^+^ T cells, we employed small molecule inhibitors of different cell death pathways. WT (*Fto*^fl/fl^CD4-Cre^−^) and FTO KO (*Fto*^fl/fl^CD4-Cre^+^) CD8^+^ T cells were stimulated in vitro with anti-CD3/CD28 antibodies in the presence of the necroptosis inhibitor necrostatin-1 (NEC-1) [37], the pyroptosis inhibitor VX-765 [38], the ferroptosis inhibitor ferrostatin-1 (Fer-1) [39] and the apoptosis inhibitor Z-VAD-FMK (Z-VAD) [40]. Interestingly, NEC-1, VX-765, and Fer-1 could not rescue the impaired CD8^+^ T cell survival caused by FTO ablation (Supplementary Fig. 5A–C). Notably, the apoptosis inhibitor Z-VAD significantly restored the upregulated apoptosis in FTO KO CD8^+^ T cells (Fig. 3A). Genome-wide transcriptomic analysis (RNA-seq) was performed to compare the transcriptomic signatures between WT and FTO KO OT-1^+^CD8^+^ T cells isolated from recipient mice 5 days after *LM*-OVA infection in the in vivo co-transfer model (Fig. 1C). We found that 522 and 286 genes were upregulated and downregulated (fold-change greater than 2, *P*-value < 0.05), respectively in FTO KO CD8^+^ T cells (Supplementary Data 1). Gene Ontology (GO) analysis of the differentially expressed genes (DEGs) from RNA-seq data revealed that in FTO-deficient CD8^+^ T cells, genes associated with programmed cell death and apoptotic process were significantly enriched while genes associated with activation of immune response and negative regulation of apoptotic process were significantly downregulated compared to WT controls (Fig. 3B). Gene set enrichment analysis (GSEA) of the hallmark pathways identified enriched gene sets involved in cell survival pathways, such as TNF signaling, p53 pathway, MYC targets and apoptosis in KO CD8^+^ T cells (Fig. 3C).

Extrinsic apoptotic pathways triggered by death receptors (e.g., Fas) can activate the intrinsic apoptotic pathway involving mitochondrial and DNA damages [41]. In line with this notion, the expression of the anti-apoptotic protein Bcl-2 was remarkably decreased in FTO KO (*Fto*^fl/fl^CD4-Cre^+^OT-1^+^) CD8^+^ T cells obtained from the in vivo co-transfer model on days 4 and 5, compared to WT (*Fto*^fl/fl^CD4-Cre^−^OT-1^+^) controls (Fig. 3D). And the expression of cleaved caspase-3, a hallmark of apoptosis, was also remarkably elevated in FTO-deficient CD8^+^ T cells stimulated in vitro with OVA_257-264_ peptide (OT-1^+^CD8^+^ T cells) (Fig. 3E) and anti-CD3/CD28 antibodies (WT CD8^+^ T cells) (Fig. 3F). Meanwhile, the downregulation of Bcl-2 and upregulation of cleaved caspase-3 were further verified by Western blot in WT and FTO-deficient CD8^+^ T cells stimulated with anti-CD3/CD28 antibodies (Fig. 3G). Together, these data indicate that the significantly reduced FTO-deficient CD8^+^ T cells during immune response are attributed to cell apoptosis.

### FTO regulates CD8^+^ T cell survival via m^6^A modification on Fas

**Fig. 4:**
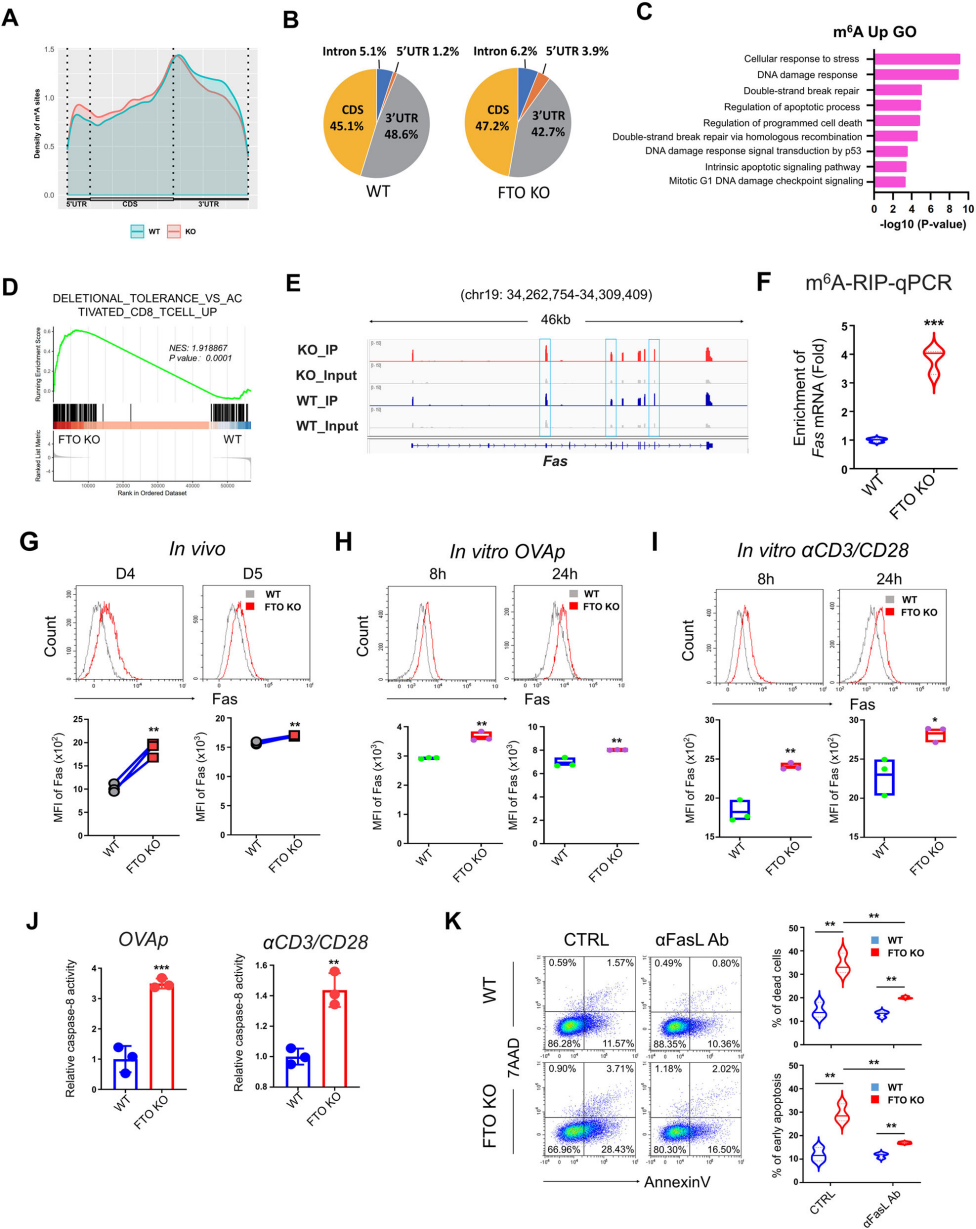
FTO regulates CD8^+^ T cell survival via m^6^A modification on Fas. **A** The metagene profile of m^6^A sites distributed across a normalized transcript segment containing three regions: 5′ UTR, CDS, and 3′ UTR in WT and FTO KO CD8^+^ T cells. **B** Pie charts showing the percentages of total m^6^A modifications distributed in 5′ UTR, CDS, intron, and 3′ UTR regions of WT and FTO KO CD8^+^ T cells. **C** GO analysis of enriched pathways using m^6^A upregulated genes in FTO KO CD8^+^ T cells compared to WT control. **D** GSEA plot depicting the enriched pathway in FTO KO CD8^+^ T cells using C7 immunologic gene sets. **E** Integrative Genomics Viewer (IGV) tracks displaying the m^6^A modification sites on *Fas* mRNA based on MeRIP-Seq data in WT and FTO KO CD8^+^ T cells. The upregulated m^6^A modification sites with high confidence were marked in cyan squares. **F** The m^6^A enrichment of *Fas* mRNA in WT and FTO KO CD8^+^ T cells was measured by m^6^A-RIP-qPCR analysis (*n* = 3). **G** Representative flow cytometry plots and the MFI of Fas expression on the WT and FTO KO donor-derived (OT-1^+^) CD8^+^ T cells in the spleen from recipient mice 4 (left) and 5 (right) days post *LM*-OVA infection in the in vivo co-transfer model (*n* = 3). **H** Representative flow cytometry plots and the MFI of Fas expression on the WT (*Fto*^fl/fl^CD4-Cre^−^OT-1^+^) and FTO KO (*Fto*^fl/fl^CD4-Cre^+^OT-1^+^) CD8^+^ T cells stimulated in vitro with OVA_257-264_ peptide for 8 h (left) and 24 h (right) (*n* = 3). **I** Representative flow cytometry plots and the MFI of Fas expression on the WT (*Fto*^fl/fl^CD4-Cre^−^) and FTO KO (*Fto*^fl/fl^CD4-Cre^+^) CD8^+^ T cells stimulated in vitro with anti-CD3/CD28 antibodies for 8 h (left) and 24 h (right) (*n* = 3). **J** Caspase-8 activity in WT and FTO KO CD8^+^ T cells stimulated in vitro with OVA_257-264_ peptide (left, OT-1^+^CD8^+^ T cells) and anti-CD3/CD28 antibodies (right, WT CD8^+^ T cells) for 24 h (*n* = 3). **K** Flow cytometry analysis of Annexin V and 7AAD expression on WT (*Fto*^fl/fl^CD4-Cre^−^) and FTO KO (*Fto*^fl/fl^CD4-Cre^+^) CD8^+^ T cells stimulated in vitro with anti-CD3/CD28 antibodies in the presence or absence of anti-FasL blocking antibodies for 24 h (*n* = 3). Data are representative of two or three independent experiments shown as the mean ± SD. Statistical testing is depicted as two-sided, unpaired *t*-tests or two-way ANOVA; *P ≤ 0.05, **P ≤ 0.01, ***P ≤ 0.001.

FTO, as one critical m^6^A demethylase, plays vital roles in a variety of biological processes through regulating the mRNA m^6^A levels and expression of target genes [22]. To identify the underlying mechanisms by which FTO controls CD8^+^ T cell survival during immune response, we performed m^6^A-methylated RNA immunoprecipitation sequencing (MeRIP-seq) to map the m^6^A landscape in WT and FTO KO CD8^+^ T cells obtained from the recipient mice 5 days after *LM*-OVA infection in the in vivo co-transfer model. The m^6^A sites distribution was displayed across the transcript regions 5′ UTR, coding sequence (CDS) and 3′ UTR. The density of m^6^A peaks was significantly enriched in the 3′ UTR region with slightly decreased in abundance at the 3′ UTR and increased abundance at the 5′ UTR and CDS regions in FTO KO groups (Fig. 4A, B). Comparing the differentially expressed methylation peaks, there were 581 and 3241 hyper-methylated and hypo-methylated (fold-change greater than 1.5, *P*-value < 0.05) transcripts, respectively, in FTO KO CD8^+^ T cells (Supplementary Data 2). Given that loss of FTO causes an upregulation of m^6^A level, we focused specifically on those hyper-methylated transcripts. GO term analysis of those transcripts uncovered a significant enrichment in pathways associated with stress response, DNA damage, programmed cell death and apoptotic signaling (Fig. 4C). In addition, pathway enrichment analysis of RNA-seq data based on C7 immunologic gene sets revealed that the deletional tolerance-associated apoptotic pathway was enriched in FTO-deficient CD8^+^ T cells (Fig. 4D). It is well-appreciated that T cell apoptosis mediated by death receptors—the most well-studied one is Fas (also known as CD95)—represents a key mechanism for periphery immunological tolerance, a process known as activation-induced cell death (AICD) [42]. Notably, among those genes in deletional tolerance pathway with m^6^A hyper-methylation, the *Fas* mRNA was highly marked by m^6^A modification at CDS regions visualized by Integrative Genomics Viewer (IGV) (Fig. 4E). Further, the m^6^A-RIP-qPCR results also confirmed that the m^6^A level on *Fas* mRNA was substantially increased in FTO-deficient CD8^+^ T cells (Fig. 4F), indicating that FTO modulates the m^6^A methylation on *Fas* mRNA in CD8^+^ T cells upon antigen activation.

Accordingly, the Fas expression was significantly elevated in FTO-deficient OT-1^+^CD8^+^ T cells, compared to WT counterparts, in the in vivo co-transfer model on days 4 and 5 post-infection (Fig. 4G). Similar results were also observed in FTO-deficient CD8^+^ T cells stimulated in vitro with both OVA_257-264_ peptide (OT-1^+^CD8^+^ T cells) (Fig. 4H) and anti-CD3/CD28 antibodies (WT CD8^+^ T cells) (Fig. 4I) for 8 h and 24 h. It is well-appreciated that the extrinsic apoptosis pathways, induced by death receptor (i.e., Fas), lead to the recruitment and activation of caspase-8, which in turn directly cleaves caspase-3 and thereby induces cell apoptosis [41, 43]. Hence, we next measured the caspase-8 activity in WT and FTO CD8^+^ T cells upon antigen stimulation. In both OT-1^+^ and WT CD8^+^ T cells, stimulated in vitro with OVA_257-264_ peptide and anti-CD3/CD28 antibodies respectively, FTO deficiency led to markedly elevated caspase-8 activity compared to WT groups (Fig. 4J). To provide further evidence of upregulated Fas impairing CD8^+^ T cell survival, we examined the cell apoptosis in WT and FTO KO CD8^+^ T cells stimulated in vitro with anti-CD3/CD28 antibodies in the presence or absence of anti-FasL blocking antibodies. As expected, Fas signaling blockade significantly mitigated the upregulated cell death and early apoptosis caused by FTO deficiency in activated CD8^+^ T cells (Fig. 4K). Furthermore, to evaluate the impact of FTO on endogenous CD8^+^ T cell responses, we conducted a direct infection model on WT and FTO conditional KO mice. WT (Fto^fl/fl^CD4-Cre^−^) and FTO KO (Fto^fl/fl^CD4-Cre^+^) mice were infected by intravenous injection of *LM* that expresses the GP33 peptide (*LM*-GP33) [44], a well-characterized CD8^+^ T cell epitope derived from the glycoprotein of lymphocytic choriomeningitis virus (LCMV) (Supplementary Fig. 6A). 7 days post-infection, the frequency and cell number of both total CD8^+^ T cells (Supplementary Fig. 6B) as well as antigen-specific tetramer-GP33^+^ CD8^+^ T cells (Supplementary Fig. 6C) were remarkably reduced in FTO KO mice compared to WT counterparts. In addition, the cell apoptosis (Supplementary Fig. 6D) and Fas expression (Supplementary Fig. 6E) were also upregulated in FTO KO CD8^+^ T cells. Together, these data suggest that FTO directly demethylates m^6^A on *Fas* mRNA; thus, FTO ablation leads to an upregulation of m^6^A modification and subsequent Fas expression, which promotes cell apoptosis in FTO KO CD8^+^ T cells upon antigen stimulation.

### Increased m^6^A methylation on *Fas* mRNA enhances its stability

**Fig. 5:**
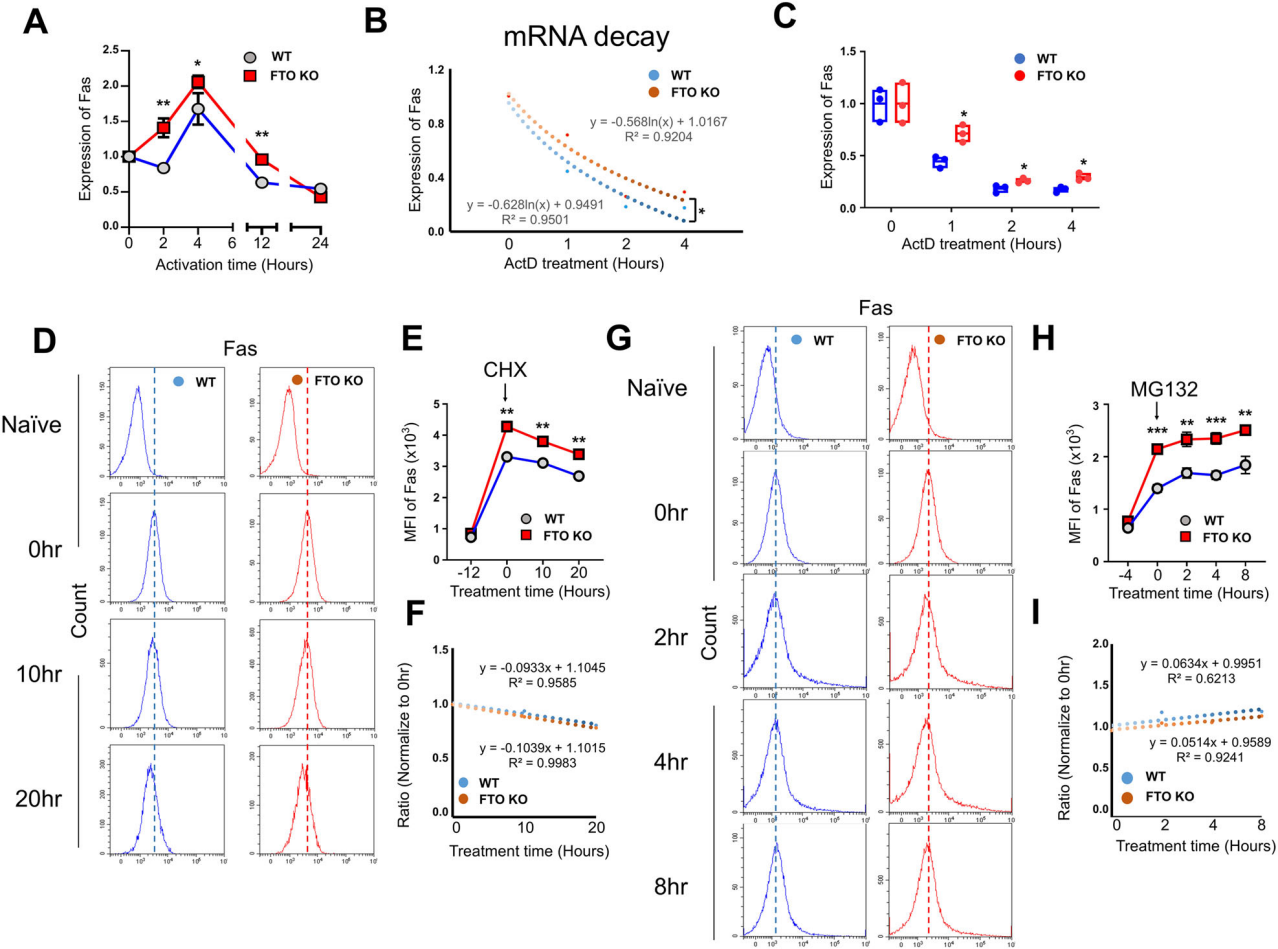
FTO deficiency enhances *Fas* mRNA stability in CD8^+^ T cells. **A** The mRNA expression of *Fas* in WT (*Fto*^fl/fl^CD4-Cre^−^) and FTO KO (*Fto*^fl/fl^CD4-Cre^+^) CD8^+^ T cells stimulated in vitro with anti-CD3/CD28 antibodies at indicated timepoints was measured by qPCR (*n* = 3). **B** mRNA decay assay showing the remaining content of *Fas* mRNA measured by qPCR in WT and FTO KO CD8^+^ T cells with ActD treatment. The remaining mRNAs were normalized to 0 h (*n* = 3). **C** The mRNA levels of Fas in (**B**) at different timepoints after ActD treatment (*n* = 3). **D**–**F** WT and FTO KO CD8^+^ T cells were stimulated with anti-CD3/CD28 antibodies for 12 h before adding in CHX treatment at different timepoints (*n* = 3). The representative flow cytometry plots (**D**) and MFI (**E**) of Fas expression on WT and FTO KO CD8^+^ T cells. CHX administration was marked with the arrow at 0 h, and naïve cells were indicated as −12 h. **F** The Fas expression on WT and FTO KO CD8^+^ T cells after CHX treatment was normalized to 0 h. **G**–**I** WT and FTO KO CD8^+^ T cells were stimulated with anti-CD3/CD28 antibodies for 4 h before adding in MG132 treatment at different timepoints (*n* = 3). The representative flow cytometry plots (**G**) and MFI (**H**) of Fas expression on WT and FTO KO CD8^+^ T cells. MG132 administration was marked with the arrow at 0 h, and naïve cells were indicated as −4 h. **I** The Fas expression on WT and FTO KO CD8^+^ T cells after MG132 treatment was normalized to 0 h. Data are representative of two or three independent experiments shown as the mean ± SD. Statistical testing is depicted as two-sided, unpaired *t*-tests; *P ≤ 0.05, **P ≤ 0.01, ***P ≤ 0.001.

Given that m^6^A methylation on mRNA modulates gene expression through influencing mRNA stability and translation efficiency [10], we next sought to explore whether the upregulated m^6^A modification on *Fas* mRNA affects its mRNA stability and translation. We first detected the mRNA levels of *Fas* in WT and FTO KO CD8^+^ T cells stimulated with anti-CD3/CD28 antibodies in vitro for different periods of time. The qPCR data showed that FTO-deficient CD8^+^ T cells had augmented mRNA expression of *Fas* along with the stimulation compared to WT cells (Fig. 5A). RNA decay assay was further performed by administration of actinomycin D (ActD), a transcription inhibitor, during CD8^+^ T cell stimulation to evaluate the stability of *Fas* mRNA. We found that the *Fas* mRNA had decelerated decay kinetics in FTO-deficient CD8^+^ T cells (Fig. 5B, C), suggesting an enhancement of *Fas* mRNA stability upon FTO ablation. To explore the effects of protein translation and degradation, we next applied protein-synthesis inhibitor cycloheximide (CHX) and proteasome inhibitor MG132 during CD8^+^ T cell activation. WT and FTO KO naïve CD8^+^ T cells were stimulated in vitro with anti-CD3/CD28 antibodies for 12 h before adding CHX to block protein synthesis. In agreement with the above results, Fas expression was rapidly elevated upon TCR stimulation, and FTO-deficient CD8^+^ T cells exhibited markedly increased Fas levels (Fig. 5D, E). CHX treatment led to a downregulation of Fas expression in both WT and KO groups (Fig. 5D, E). However, the decline of Fas expression after CHX treatment was comparable between WT and KO cells when comparing to its original expression level at 0 h (before CHX treatment) (Fig. 5F). Additionally, WT and FTO KO naïve CD8^+^ T cells were stimulated with anti-CD3/CD28 antibodies for 4 h before adding in MG132 to block protein degradation. The results showed that FTO-deficient CD8^+^ T cells displayed significantly enhanced Fas expression upon stimulation irrelative with MG132 treatment (Fig. 5G, H). Similarly, the dynamic expression of Fas after MG132 was comparable between WT and KO cells (Fig. 5I). All these data suggest that FTO ablation promotes Fas expression via enhancing the *Fas* mRNA m^6^A methylation and its stability.

### FTO modulates Fas expression dependent on m^6^A reader IGF2BP3

**Fig. 6:**
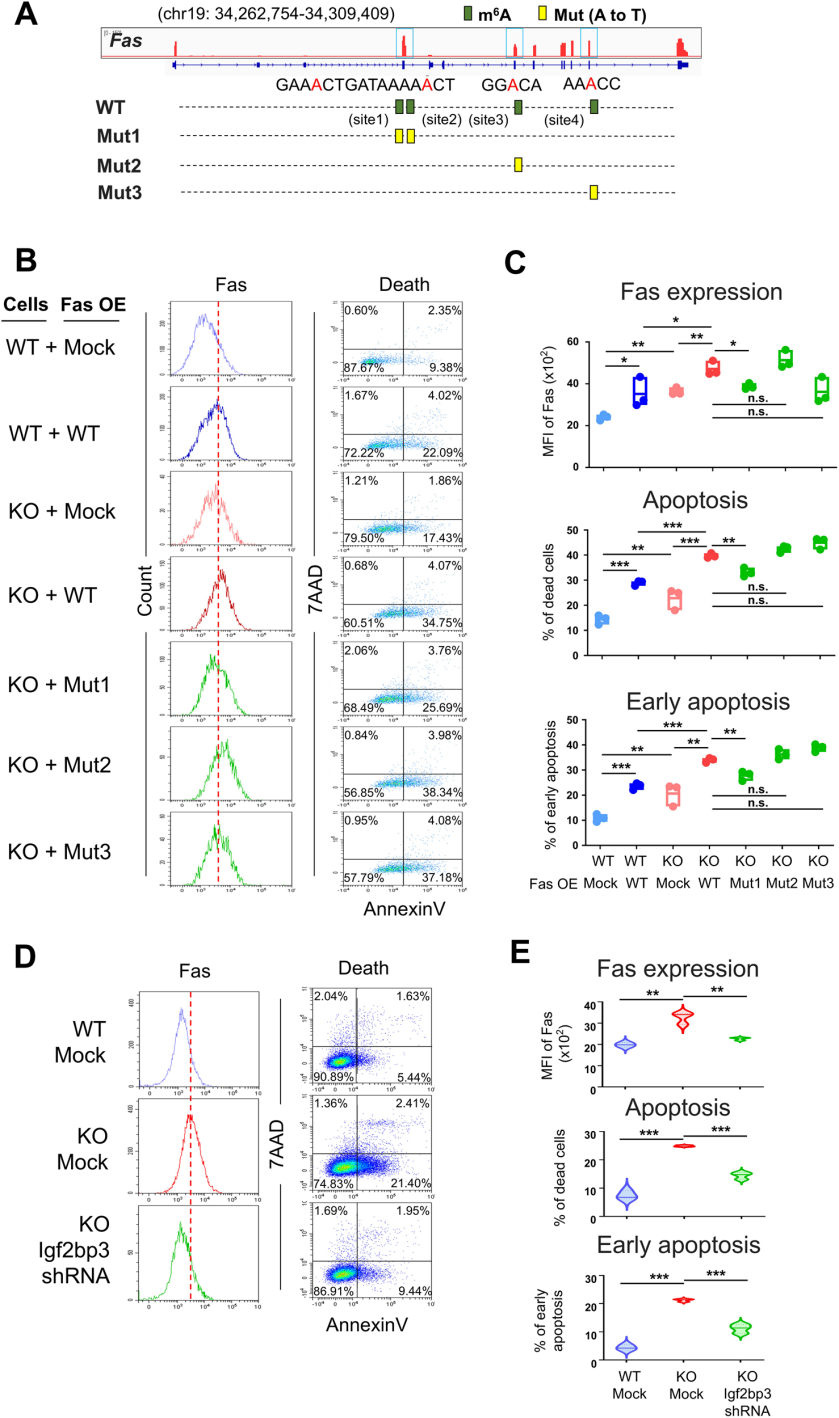
Loss of FTO enhances the IGF2BP3-mediated transcription of Fas. **A** Schematic representation of plasmid constructs with WT and m^6^A site mutations on *Fas* mRNA (mut 1: mutations on both site 1 and site 2; mut 2: mutation on site 3; mut 3: mutation on site 4). **B** Representative flow cytometry plots showing Fas expression and Annexin V/7AAD expression on WT (*Fto*^fl/fl^CD4-Cre^−^) and FTO KO (*Fto*^fl/fl^CD4-Cre^+^) CD8^+^ T cells overexpressed (OE) with Mock, *Fas* WT or mutant plasmids followed by stimulation with anti-CD3/CD28 antibodies for 24 h (for Fas detection) and 48 h (for Annexin V/7AAD detection) (*n* = 3). **C** Fas expression levels, frequencies of total dead cells, and early apoptotic cells were shown in different groups of CD8^+^ T cells from (**B**). n.s. means not significant. **D** Representative flow cytometry plots showing Fas expression and Annexin V/7AAD expression on WT (*Fto*^fl/fl^CD4-Cre^−^) and FTO KO (*Fto*^fl/fl^CD4-Cre^+^) CD8^+^ T cells transfected with either Mock or *Igf2bp3* shRNA followed by stimulation with anti-CD3/CD28 antibodies for 24 h (for Fas detection) and 48 h (for Annexin V/7AAD detection) (*n* = 3). **E** Fas expression levels, frequencies of total dead cells, and early apoptotic cells were shown in different groups of CD8^+^ T cells from (**D**). Data are representative of two or three independent experiments shown as the mean ± SD. Statistical testing is depicted as two-sided, unpaired *t*-tests or one-way ANOVA; *P ≤ 0.05, **P ≤ 0.01, ***P ≤ 0.001.

To validate the FTO regulation of Fas expression dependent on m^6^A modification, we analyzed the m^6^A methylation sites on *Fas* mRNA based on the conserved m^6^A motif RRACH (R = G or A; H = A, C, or U) [45], and yielded four upregulated m^6^A sites within the CDS region in FTO-deficient CD8^+^ T cells (Fig. 6A). We then generated constructs containing both WT and mutant *Fas* with m^6^A methylation sites mutation. Owing to a close proximity (within 10 bp) between the first two sites (shown in IGV), we constructed three *Fas* mutants with mutations at both site 1 and 2 (mutant 1), site 3 (mutant 2) and site 4 (mutant 3) (Fig. 6A). As anticipated, overexpression of WT Fas in both WT and FTO KO CD8^+^ T cells could significantly elevate the Fas expression and cell apoptosis compared to that overexpressed with the empty vector (Mock) (Fig. 6B, C, first four panels). More noticeably, overexpression of WT Fas in FTO KO CD8^+^ T cells, compared to WT CD8^+^ T cells, resulted in even higher levels of Fas expression and cell apoptosis (Fig. 6B, C, first four panels). Notably, transfection with both Fas WT and mutants resulted in comparable Fas expression levels (Supplementary Fig. 7A). However, overexpression of *Fas* mutant 1, but not mutant 2 and 3, into FTO KO CD8^+^ T cells significantly decreased the Fas expression, total cell apoptosis as well as early apoptosis levels (Fig. 6B, C, last three panels), suggesting that FTO likely regulates CD8^+^ T cell apoptosis through modifying the m^6^A methylation on *Fas* mRNA at site 1 and 2.

Furthermore, m^6^A modification is a dynamic process in which the methyltransferases and demethylases mainly control the m^6^A levels on target transcripts, whereas the RNA binding proteins (RBPs), namely m^6^A readers, determine the biological function of these modifications [10]. Given that IGF2BPs (IGF2BP1/2/3), among distinct types of m^6^A reader proteins, play a dominant role in modulating the stability of m^6^A-targeted mRNAs [46], we mainly focused on this family of proteins. We first measured the expression of IGF2BPs in WT and Fas KO CD8^+^ T cells isolated from recipient mice 5 days after infection in the in vivo co-transfer model by qPCR, and found that IGF2BP3, but not IGF2BP1 and IGF2BP2, had enhanced expression in KO cells, suggesting it might play a regulatory role in FTO ablated CD8^+^ T cells (Supplementary Fig. 7B). In addition, according to the m^6^A2Target website (http://rm2target.canceromics.org), a comprehensive database containing m^6^A writer, eraser, and reader target genes, IGF2BP3 has been shown to bind to *Fas* with m^6^A modification (Supplementary Table 1) [47]. To validate its role in mediating the upregulation of Fas in FTO-deficient CD8^+^ T cells, the knockdown of *Igf2bp3* using shRNA was performed. Transduction of *Igf2bp3* shRNA into WT CD8^+^ T cells could efficiently downregulate IGF2BP3 expression assessed by qPCR (Supplementary Fig. 7C). When *Igf2bp3* shRNA was introduced into FTO KO CD8^+^ T cells, compared to WT and FTO KO CD8^+^ T cells transduced with Mock, the knockdown of *Igf2bp3* significantly reduced the enhanced Fas expression and cell apoptosis caused by FTO ablation (Fig. 6D, E). Collectively, these data suggest that FTO-mediated m^6^A methylation regulates Fas expression in activated CD8^+^ T cells in a manner dependent on m^6^A reader protein IGF2BP3.

## Discussion

**Fig. 7:**
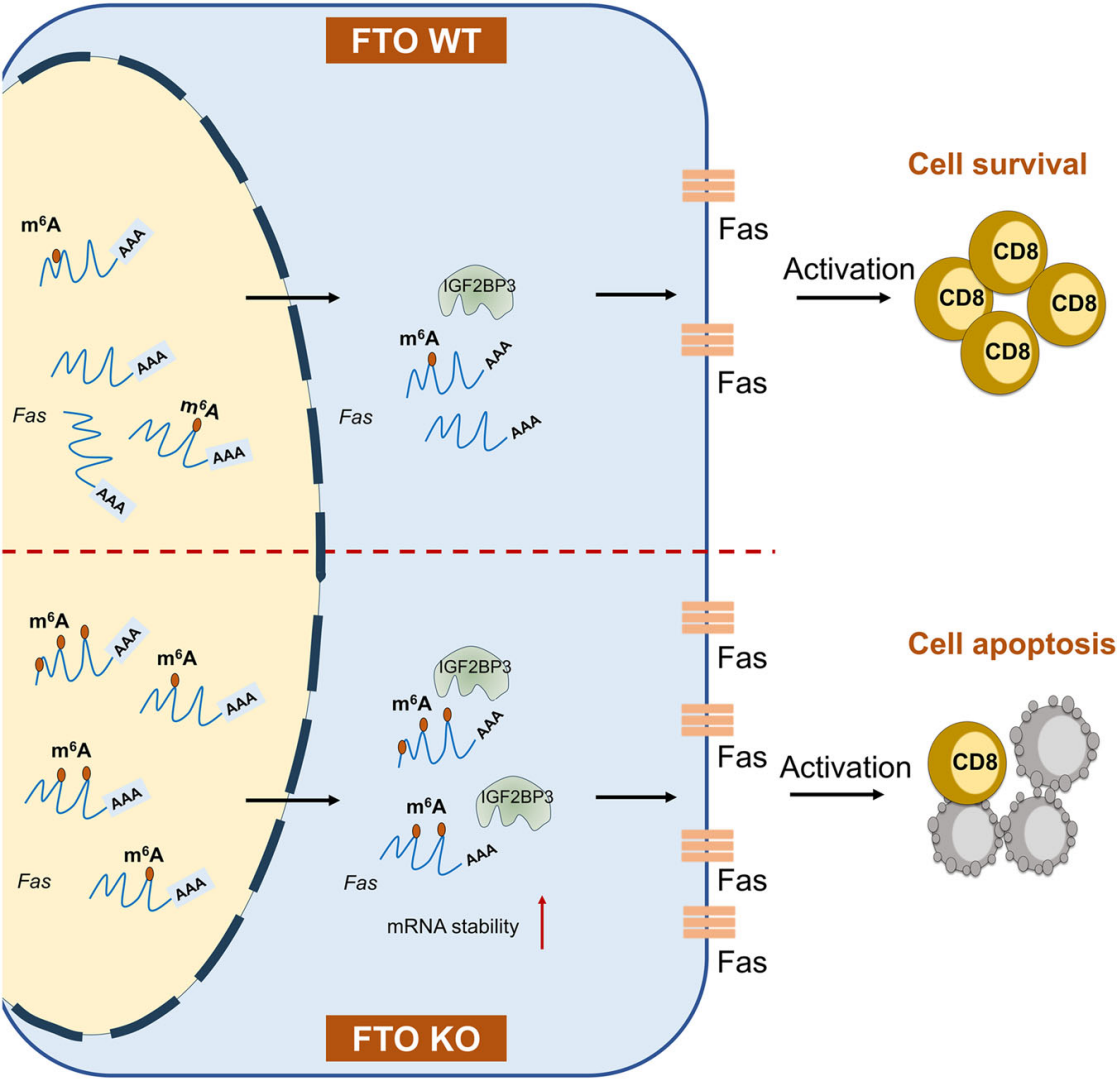
Mechanistic scheme showing the FTO-mediated m^6^A methylation of Fas in regulating CD8^+^ T cell survival and effector response. In WT CD8^+^ T cells, the presence of FTO effectively demethylates m^6^A modification on *Fas* mRNA, which leads to appropriate Fas expression and cell survival upon antigen stimulation. Compared to WT cells, FTO-deficient CD8^+^ T cells exhibit elevated m^6^A modification on *Fas* mRNA, which promotes its mRNA stability dependent on m^6^A reader protein IGF2BP3, resulting in subsequent increases of Fas expression and cell apoptosis upon cell activation.

Understanding the underlying regulatory mechanisms involved in CD8^+^ T cell immune responses is profoundly essential in protecting against infection and tumors, as well as offering potential opportunities in the clinical development of T cell based immunotherapy [48]. Mechanisms regulating CD8^+^ T cell differentiation and functionality during infections at the transcriptional, epigenetic (DNA and histone modification), and metabolic levels have been extensively characterized [2, 4]. Epigenetic regulations at the RNA level, such as RNA m^6^A methylation, have gained emerging attention for their essential roles in a variety of biological and pathological conditions [10]. In this study, we investigated the effects of m^6^A modification in CD8^+^ T cell immune responses by targeting the m^6^A demethylase FTO. We unveiled that FTO is indispensable for maintaining CD8^+^ T cell survival, expansion, and immunological function during acute infection. Our data revealed that T cell-specific deficiency in FTO led to an elevation of apoptosis in CD8^+^ T cells upon antigen stimulation. Loss of FTO resulted in an upregulation of m^6^A modification on *Fas* mRNA, which promoted the mRNA stability dependent on m^6^A reader IGF2BP3. The increased mRNA and consequent protein expression of Fas triggered the apoptotic process in activated CD8^+^ T cells (Fig. 7).

Emerging evidence has demonstrated that m^6^A modification plays a key role in regulating the immunological functions of T cells, yet the majority of early studies focused on CD4^+^ T cell homeostasis and differentiation [49]. Recently, Guo et al. investigated the intrinsic role of m^6^A methyltransferase METTL3 in CD8^+^ T cell responses during acute viral infection [18]. They reported that METTL3-deficient CD8^+^ T cells exhibited impaired effector cell differentiation, defective cell survival, cytokine production, and subsequent memory formation, which relied on the regulatory mechanism of METTL3-m^6^A-T-bet axis [18]. Interestingly, our study, by targeting m^6^A demethylase FTO in T cells, revealed significantly different phenotypes in CD8^+^ T cells during acute infection. Our results elucidated that FTO ablation led to a dramatic CD8^+^ T cell apoptosis attributed to the upregulated Fas expression upon antigen stimulation. However, FTO deficiency had limited effects on CD8^+^ T cell proliferation, cytokine production, and effector cell differentiation. These divergent data explicitly suggest that m^6^A methyltransferases and demethylases regulate distinct biological events or functions, likely through modifying distinct genes, even in the same type of cells. Similar results were observed in m^6^A regulation of Th17 cell differentiation. METTL3-deficient naïve CD4^+^ T cells showed less differentiation into Th17 cells, whereas ALKBH5 deficiency in CD4^+^ T cells also resulted in impaired IL-17 signaling [35]. METTL3 or METTL14 knockdown in CD4^+^ T cells could promote Tfh cell development upon LCMV infection [50]. However, other studies reported that METTL3 ablation in CD4^+^ T cells had impaired Tfh cell differentiation upon LCMV infection [17]. Moreover, METTL3 deficiency induced cell apoptosis in CD8^+^ T cells [18] but not in CD4^+^ T cells upon TCR stimulation [15]. Distinct phenotypes could be observed in CD8^+^ T cells with METTL3 ablation driven by different Cre promoter (*Cd4*-Cre versus *Gzmb*-Cre), which was related to different T cell activation status [18]. All these findings strongly indicate that m^6^A modification is a biological context-dependent process, highly associated with enzymatic activity, cell types, and immunological environment.

Since FTO was first discovered to be associated with fat mass and obesity, a large number of studies have demonstrated that FTO plays critical roles in metabolic diseases, such as obesity and diabetes, and cardiovascular diseases [51, 52]. FTO modulates adipocyte biology by promoting differentiation, adipogenesis and lipid metabolism [52, 53]. FTO inhibits insulin secretion in pancreatic β cells through NF-κB activation [54, 55]. Emerging recent evidence has revealed the essential function of FTO in promoting tumorigenesis, and FTO can serve as a potential cancer therapeutic target [24, 56]. FTO exerts oncogenic effects on various types of cancer cells, promoting tumor cell growth by activating survival and cell-cycle signaling, such as PI3K/Akt/mTOR pathway, MYC, β-catenin, and CEBPA [10, 57]. However, the regulatory roles of FTO in immune cells are much less studied. A few evidence has revealed that FTO, through meditating m^6^A methylation, regulates differentiation and function in macrophages and NK cells [25–28]. These findings indicate that FTO-mediated m^6^A regulates gene expression in cell type- and biological context-dependent manners. In this study, we evaluate the function of FTO in CD8^+^ T cells in the adaptive immune system. FTO plays a pivotal role in maintaining cell survival in CD8^+^ T cells during immune responses through controlling the m^6^A levels and mRNA stability of the death receptor Fas. This data underscores the special mechanism of FTO-mediated m^6^A modification on apoptosis is uniquely pronounced in CD8^+^ T cells.

As a member of the TNFR superfamily of death receptors, Fas interacts with FasL, initiating a complex cascade of cell apoptosis and playing important roles in T cell immune responses [58]. Apoptosis is primarily mediated by two classical pathways: the extrinsic pathway triggered by death receptors (e.g., Fas) and the intrinsic pathway involving mitochondrial and DNA damages [41, 59]. Extrinsic apoptotic pathways can activate the intrinsic apoptotic pathway, linking death receptor signaling to mitochondrial apoptosis. Activation of Fas/FasL signaling recruits FADD, leading to caspase-8 activation. Active caspase-8 cleaves Bid into tBid, which translocates to mitochondria and inhibits anti-apoptotic Bcl-2 proteins, thereby initiating the intrinsic pathway, caspase-3 cleavage, and final cell apoptosis [41, 60]. In this study, we observed upregulated Fas expression, increased caspase-8 activity, which is the primary downstream effector of Fas/FasL signaling, elevated caspase-3 activity, and enhanced cell apoptosis, accompanied by reduced Bcl-2 levels in FTO KO CD8^+^ T cells. These findings indicate that the interplay between extrinsic and intrinsic apoptotic pathways cooperatively contributes to the cell apoptosis of FTO-deficient CD8^+^ T cells. Furthermore, Fas-triggered T cell apoptosis depends on TCR ligation, which increases Fas expression and provides additional “competency signal” to ensure antigen-specific T cell apoptosis [61]. However, TCR stimulation does not elicit immediate T cell death [62]. The underlying mechanism was suggested to be associated with a few days-delay of surface expression of Fas or FasL after TCR ligation [63]. Besides regulation by gene transcription and translation, we here found that Fas expression was governed by a novel epigenetic regulation of RNA m^6^A modification. Under the physiological condition, TCR stimulation induces *Fas* gene transcription with a balanced m^6^A modification on *Fas* mRNA, which results in a gradual increase of Fas expression during the first few days of CD8^+^ T cell activation. The upregulated Fas expression subsequently induces apoptosis to avoid T cell overactivation [43]. However, with the ablation of m^6^A eraser FTO, the m^6^A levels on *Fas* mRNA were significantly increased, which promoted its mRNA stability. Therefore, the elevated m^6^A modification on *Fas* mRNA induces an accelerated elevation of Fas expression and sensitivity to Fas-induced apoptosis during CD8^+^ T cell immune response.

The m^6^A methylation is dynamically controlled by methyltransferases and demethylases, whereas the eventual biological functions of m^6^A modification are determined by m^6^A reader proteins. The m^6^A reader proteins recognize and bind to m^6^A-marked transcripts to mediate a variety of processes, such as mRNA stability, splicing, translocation, and translation [10]. Distinct m^6^A readers exist in different cell types and conduct biological context-dependent functions. For instance, previous work has shown that METTL3-mediated m^6^A modification exhibited discrete functions in naïve CD4^+^ T cells and Treg cells [15, 64]. m^6^A reader proteins can be classified into three categories: YTH family proteins, HNRNP family proteins and IGF2BP family proteins [11]. IGF2BPs are recently identified an RBP family as m^6^A readers, mainly promoting the stability and storage of their target mRNAs [65]. Our data demonstrate that FTO ablation leads to increased m^6^A methylation on *Fas* mRNA, which promotes Fas expression via enhancing the *Fas* mRNA stability. Therefore, in the investigation of m^6^A readers that mediate FTO’s regulation of *Fas* mRNA stability, we mainly focused on IGF2BP family members. IGF2BPs have been shown to play critical roles in promoting cancer cell growth and creating immunosuppressive tumor microenvironment in various types of cancer [66]. However, the direct regulatory roles of IGF2BPs in T cells are currently undefined. Here, we found that IGF2BP3 mediated the enhanced *Fas* mRNA stability in FTO-deficient CD8^+^ T cells, thus, knockdown IGF2BP3 significantly decreased the Fas expression and cell apoptosis caused by FTO ablation. These findings suggest that the m^6^A-mediated regulation in CD8^+^ T cells is a sophisticated process orchestrated by the full m^6^A machinery of readers, erasers, and readers.

The current findings of FTO regulating m^6^A modification on *Fas* mRNA through IGF2BP3 are supported by the following pieces of evidence: [1] MeRIP-seq and MeRIP-PCR data identifying upregulated m^6^A peaks on *Fas* mRNA in FTO-deficient CD8^+^ T cells, which is consistent with the demethylase activity of FTO [2]. Site-directed mutagenesis confirms the functional relevance of FTO-mediated m^6^A methylation in Fas expression and apoptosis regulation [3]. The public database (m^6^A2Target website) demonstrates a direct IGF2BP3 binding to *Fas* mRNA depending on m^6^A. However, more direct experimental evidence of FTO/IGF2BP3 binding to m^6^A sites of *Fas* mRNA, such as CLIP-PCR, biotin-labeled RNA pull-down, and RNA binding protein immunoprecipitation (RIP), would further strengthen this study. In addition, as a critical m^6^A demethylase, FTO is broadly expressed and exerts diverse regulatory roles in diverse biological processes depending on different cellular contexts [24, 67]. Here, we found that FTO ablation in CD8^+^ T cells mostly led to increased apoptosis. Thus, to identify FTO targets, we primarily focused on cell death/apoptosis-related genes in the MeRIP-seq data. However, the m^6^A levels of majority of those genes, such as *Bcl-2*, *Myc*, *Faslg*, *Fadd*, *Bcl2l1* (Bcl-xl), *Cflar* (cFLIP), *Bax*, *Bak*, *Casp3*, *Casp7*, and *Casp8*, were not upregulated, while *Fas* was emerged as a key candidate in FTO KO CD8^+^ T cells. While we acknowledge that FTO may also influence CD8^+^ T cell immune responses indirectly through other signaling pathways, further systematic exploration of FTO’s broader regulatory network, for example, employing genome-wide CRISPR screens and multi-omics approaches, is required in future research.

In conclusion, our study defines a novel mechanism of FTO-mediated m^6^A modification in maintaining CD8^+^ T cell survival upon antigen stimulation, providing new insights into understanding the RNA-based epigenetic regulation in CD8^+^ T cell immune responses. Notably, the discrepant findings observed in FTO and METTL3-deficient CD8^+^ T cells evidently suggest that m^6^A readers and erasers have markedly distinct downstream genes. It will be intriguing to explore the exact regulatory mechanisms that m^6^A readers and erasers employ in CD8^+^ T cells in the future.

## Materials and methods

### Mice

*Fto*^fl/fl^, CD4*-*Cre ^+^, CD45.1^+^, and OT-1 strains were purchased from the Jackson Laboratory (Bar Harbor, ME, USA). *Fto*^fl/fl^ mice were crossed with CD4*-*Cre and OT-1 mice to generate *Fto*^fl/fl^CD4*-*Cre^*+*^(OT-1^+^) (KO) and *Fto*^fl/fl^CD4*-*Cre^−^(OT-1^+^) (WT) mice. CD45.1^+^ mice were crossed with OT-1^+^ or CD45.2^+^ mice to generate OT-1^+^CD45.1^+^ or CD45.1^+^CD45.2^+^ mice. 8-12-week-old female mice were preferentially used in this study. All mice were housed in specific-pathogen free conditions by the Xi’an Jiaotong University Division of Laboratory Animal Research.

### Antibodies and flow cytometry

Single-cell preparations were stained with the monoclonal antibodies purchased from Biolegend (San Diego, CA, USA): anti-mouse CD45.1 (#110716, clone A20), anti-mouse CD45.2 (#109806, clone 104), anti-mouse CD3E (#100334, clone 145-2C11), anti-mouse CD8A (#100725, clone 53-6.7), anti-mouse KLRG1 (#138412, clone 2F1/KLRG1), anti-mouse IL-7R (#135014, clone A7R34), anti-mouse CD44 (#103028, clone IM7), anti-mouse CD62L (#104412, clone MEL-14), anti-mouse CD25 (#102030, clone PC61), anti-mouse CD69 (#104508, clone H1.2F3), anti-mouse IL-2 (#503820, clone JES6-5H4), anti-mouse IFN-γ (#505826, clone XMG1.2), anti-mouse TNF-α (#506308, clone Mp6-XT22), anti-mouse granzyme B (#372208, clone QA16A02), anti-mouse perforin (#154306, clone S16009a), anti-mouse T-bet (#644824, clone 4B10), anti-mouse Eomes (#157706, clone W17001A), anti-mouse Bcl-2 (#658706, clone 100), anti-mouse CD95 (#152608, clone SA367H8), anti-mouse BrdU (#364116, clone 3D4) and anti-mouse Ki67 monoclonal antibody (#652422, clone 16A8). Anti-mouse active Caspase-3 monoclonal antibody (#570334, clone C92-605.rMAb) was obtained from BD Biosciences. Phorbol 12-myristate 13-acetate (PMA, # HY-18739), Ionomycin (#HY-13434), brefeldin A (#HY-16592), and monensin (#HY-N0150) were purchased from MedChemExpress.

For analysis of surface markers, cells were stained in PBS containing 1% FBS on ice for 30 min. For analysis of intracellular cytokine staining, cells were stimulated for 4 h in vitro with PMA/Ionomycin in the presence of brefeldin A and monensin. The stimulated cells were fixed and permeabilized using a Fixation/Permeabilization Solution Kit (#420801 and #421002, Biolegend). For nuclear protein staining, such as Ki67, T-bet, Eomes, and Bcl-2, cells were fixed and permeabilized using a Transcription Factor Fixation/Permeabilization Concentrate and Diluent purchased from eBioscience (#00-5523-00, San Diego, CA, USA).

For BrdU staining, cells were first stained with indicated surface markers followed by BrdU staining according to the manufacturer’s instruction (#554714, BrdU Flow Kit, BD Biosciences). For apoptosis, cells were stained with 7-Aminoactinomycin D (7AAD, #420404)/Annexin V (#640947) (Biolegend). Cells were analyzed using a CytoFLEX flow cytometer (BECKMAN COULTER). FlowJo software and CytExpert software were used for data analysis. For CD8^+^ T cell sorting, FACSAria II sorter was applied (BD Biosciences).

### Quantitative PCR (qPCR)

To examine the mRNA levels, Quick-RNA Microprep Kit (#R1051, Zymo Research) was used to extract total RNA from purified T cells according to the manufacturer’s instruction. cDNA was reverse-transcribed using the cDNA synthesis kit (#FSQ-301, TOYOBO) and amplified with SYBR Green RT-qPCR Mastermix (#A311-05, GenStar) at StepOnePlusTM Real-Time PCR System (ThermoFisher). Primers were synthesized from Tsingke Biotechnology. Primer sequences used in this study were: mouse FTO forward 5′-AAAGTTTGAAGGAGGGGAGAAGTG-3′, reverse 5′-ACCAAAGAGGGGGAGACAGTTACG-3′; mouse Fas forward 5′- AAGTCCCAGAAATCGCCTATG-3′, reverse 5′- GGTATGGTTTCACGACTGGAG-3′; mouse IGF2BP1 forward 5′- CCTGGCTCATAACAACTTCGTCG-3′, reverse 5′- CCTTCACAGTGATGGTCCTCTC-3′; mouse IGF2BP2 forward 5′- TGAAGCCTGTGCCAATGCTGAG-3′, reverse 5′- CCAGTCGAAAAGATGCCAAGTGC-3′; IGF2BP3 forward 5′- CCACCCAGTTTGTTGGAGCCAT-3′, reverse 5′- GGATAGTAATGGACTTCTCCGCG-3′; and mouse HPRT forward 5′- AGTACAGCCCCAAAATGGTTAAG-3′, reverse 5′- CTTAGGCTTTGTATTTGGCTTTTC-3′.

### Western blotting

Total proteins were extracted from CD8^+^ T cells at indicated experiments by cell lysis in RIPA buffer (#P0013B, Beyotime Biotechnology). Protein samples were separated on SDS-polyacrylamide gels and electro-transferred onto Polyvinylidene fluoride (PVDF) membranes (#IPVH00010, Millipore). Membranes were then blocked with 5% skimmed milk before incubation with indicated primary antibodies at 4 °C overnight, followed by incubation with secondary antibodies at room temperature for one hour. The protein expression was measured by Fusion-Solo.6s (VILBER). Primary antibodies used in Western blot assay were: Bcl-2 (#sc-7382, clone C-2, Santa Cruz), cleaved caspase-3 (#9664, clone 5A1E, CST) and Actin (#60008-1-Ig, clone 7D2C10, Proteintech). The secondary antibodies purchased from Cwbio were HRP goat anti-rabbit IgG (#CW0103S) and HRP goat anti-mouse IgG (#CW0102S). Uncropped western blots were included in Supplementary Data 3.

### T cell adoptive transfer and LM Infection

In the in vivo infection model, naïve OT-1^+^CD8^+^ T cells were sorted using FACSAria II (BD Bioscience) cell sorter from WT (CD45.1^+^) and KO (CD45.2^+^; *Fto*^fl/fl^CD4*-*Cre^*+*^) mice and either separately (each 1 × 10^5^) transferred or co-transferred (each 5 × 10^4^) at a ratio of 1:1 into WT recipient mice (CD45.1^+^CD45.2^+^). Twenty-four hours after cell transfer, mice were infected with 2 × 10^4^ colony-forming units (CFU) of *LM*-OVA intravenously (i.v.). The lymphocytes in peripheral blood and spleen tissues were analyzed on the indicated days post-infection. To examine in vivo proliferation, mice were injected intraperitoneally (i.p.) with 1 mg BrdU (#B5002, Sigma-Aldrich) for 4 h before cell analysis. For quantification of bacterial burden, 1 × 10^6^ CFU *LM*-OVA was applied, and spleens and livers from infected mice were homogenized in PBS. Serial dilutions of the tissue homogenate were prepared, and 50 μl of each dilution was cultured on brain-heart infusion (BHI) agar plates. After incubation overnight at 37 °C, colonies were counted, and the *LM* CFUs recovered from each tissue were calculated. In the direct infection model, WT and FTO KO mice were infected with 1 × 10^5^ CFU of *LM*-GP33 intravenously (i.v.). The lymphocytes in the spleen were analyzed 7 days post-infection. Antigen-specific CD8^+^ T cells were stained by GP33 tetramer (NIH Tetramer Core Facility, Atlanta, GA, USA).

### T cell in vitro stimulation

To examine the T cell activation, proliferation, and apoptosis in vitro, splenocytes were isolated and stimulated with 2.5 µg/ml coated anti-CD3 (#100302, clone 145-2C11, Biolegend) and 1 µg/ml soluble anti-CD28 (#102102, clone 37.51, Biolegend) antibodies. Splenocytes isolated from OT-1^+^ mice were stimulated with 1 µg/ml OVA_257-264_ peptide. The following stimulation timepoints were applied: 24 h (activation), 48 h (proliferation), or indicated timepoints (apoptosis). For BrdU incorporation, 10 µM BrdU (#B5002, Sigma-Aldrich) was added 40 min before cell analysis. To examine the cell death pathway, CD8^+^ T cells were stimulated with anti-CD3/CD28 antibodies in the presence of 10 µM NEC-1 (#HY-15760, MedChemExpress), 20 µM VX-765 (#S2228, Selleck), 10 µM Fer-1 (#SML0583, Sigma-Aldrich), 20 µM Z-VAD (#S7023, Selleck) and 10 µg/ml anti-FasL blocking antibodies (#106611, clone MFL3, Biolegend) for 48 h. To evaluate the effects of protein synthesis and degradation, CD8^+^ T cells were stimulated with anti-CD3/CD28 antibodies for 12 h and 4 h before adding in 30 µg/ml CHX (#HY-12320, MedChemExpress) and 10 µM MG132 (#HY-13259, MedChemExpress), respectively, for another indicated time periods.

### RNA decay assay

WT and FTO CD8^+^ T cells were stimulated with anti-CD3/CD28 antibodies for 4 h before treatment with the transcription inhibitor actinomycin D (5 µg/ml, # HY-17559, MedChemExpress) for another 1, 2 and 4 h before cell collection. Total RNA was isolated by using a Quick-RNA Microprep Kit (#R1051, Zymo Research). qPCR was performed as described above to quantify the relative levels of target mRNAs.

### Analysis of caspase-8 activity

WT and FTO CD8^+^ T cells were stimulated with anti-CD3/CD28 antibodies for 24 h. The caspase-8 activity was assessed by the Caspase-8 Activity Colorimetric Assay Kit (#C1151, Beyotime Biotechnology) according to the manufacturer’s protocol.

### cDNA and shRNA constructs, retroviral production, and transfection

Expression plasmids of mouse *Fas* WT and mutants, and *Igf2bp3* shRNA were constructed into MSCV-based expressing vectors, which was engineered to include a EGFP reporter gene. Mutations were introduced via site-directed mutagenesis PCR. BOSC 23 cells (from Dr. Garry Nolan Lab) [68] were used for retrovirus generation. Mycoplasma contamination of cell lines was regularly checked. Retrovirus was produced by transfecting 5 µg of each retroviral vector in combination with 1 μg pCL-Eco retrovirus packaging vector into 6-well plates of cultured BOSC 23 cells using jetPRIME transfection reagent (#101000001, Polyplus). Following 48 h and 72 h, the culture supernatant containing retrovirus was collected through 0.45 µm filters. Purified CD8^+^ T cells were stimulated overnight using plate-bound anti-CD3 (5 µg/ml) and anti-CD28 antibodies (2 µg/mL). Spin infection of stimulated T cells using retrovirus supernatant was performed at 2500 rpm for 90 min at 37 °C. EGFP^+^ cells were identified as successfully transfected cells and examined using flow cytometry analysis or sorted for further experiments. The *Igf2bp3* shRNA sequence was GAATCATCTGAAGTTCCTCAA.

### RNA-Seq library preparation and sequencing

For RNA-seq analysis, WT and FTO KO CD8^+^ T cells were isolated from recipient mice 5 days after *LM*-OVA infection in the in vivo co-transfer model. Total RNA was extracted using the RNeasy Mini Kit (#74104, Qiagen) according to the manufacturer’s protocol. RNA-seq analysis was performed by Genergy Biotechnology according to the manufacturer’s standard protocol. RNA concentration was detected by the Qubit RNA broad range assay in the Qubit Fluorometer (Invitrogen). After quality control using an RNase-free agarose gel and Agilent 2100 (Agilent Technologies, Palo Alto, CA, USA), RNA-Seq libraries were prepared using 200 ng total RNA with TruSeq RNA sample prep kit (Illumina). Oligo(dT)-enriched mRNAs were fragmented randomly with fragmentation buffer, followed by the first- and second- strand cDNA synthesis. After a series of terminal repairs, the double-stranded cDNA library was obtained through PCR enrichment and size selection. The prepared cDNA library was sequenced with the Illumina Hiseq 2000 sequencer (Illumina HiSeq 2000 v4 Single-Read 50 bp) after pooling according to its expected data volume and effective concentration. The raw reads were trimmed by using Trim Galore with default parameters. After removing low-quality bases RNAs, the clean data were mapped to mouse genome (GRCm39) using Hisat2 RNA-Seq alignment software, and unique reads were retained to quantify gene expression counts from bam files by using featurecounts. The raw counts were calculated into TPM (Transcripts per million). Data were analyzed and preprocessed in the R environment. Differential expression analysis was performed using R package DESeq2 (*P*-value < 0.05 and abs| fold-change| greater than 2). Heatmaps and bar graphs were visualized using the R package.

### MeRIP-seq

MeRIP-seq was performed at the LC Sciences, LLC, according to the manufacturer’s standard protocol. Total RNA was isolated and purified using TRIzol reagent (Invitrogen, Carlsbad, CA, USA) from WT and FTO KO CD8^+^ T cells isolated from the recipient mice 5 days after *LM*-OVA infection in the in vivo co-transfer model. m^6^A immunoprecipitation was performed according to standard procedures as described previously [69]. Total RNA integrity was assessed using a Bioanalyzer 2100 (RIN > 7.0) and denaturing agarose gel electrophoresis. Ribosomal RNA was depleted from ~25 μg of total RNA using the Epicentre Ribo-Zero Gold Kit (Illumina). The rRNA-depleted RNA was fragmented using the Magnesium RNA Fragmentation Module (NEB) at 86 °C for 7 min. Fragmented RNA was immunoprecipitated with an m6A-specific antibody (Synaptic Systems) in IP buffer at 4 °C for 2 h. The precipitated RNA was reverse-transcribed into cDNA using SuperScript™ II Reverse Transcriptase (Invitrogen). Second-strand DNA synthesis was performed with E. coli DNA polymerase I, RNase H (NEB), and dUTP. A-tailing and adapter ligation were conducted, followed by size selection using AMPureXP beads. U-labeled second strands were digested with UDG enzyme (NEB), and libraries were PCR-amplified (8 cycles). Final libraries had an average insert size of 300 ± 50 bp. m^6^A-seq was performed at the LC Sciences, LLC on an Illumina Novaseq™ 6000 platform with 2 × 150 bp paired-end sequencing (PE150).

### Bioinformatic analysis

For MeRIP-seq data analysis, sequence quality of IP and Input samples were verified using FastQC (https://www.bioinformatics.babraham.ac.uk/projects/fastqc/) and RseQC (http://rseqc.sourceforge.net/). Sequencing reads were mapped to the mouse reference genome (mm39) using HISAT2 (http://daehwankimlab.github.io/hisat2). m^6^A-enriched peaks and diff peaks were identified by exomePeak2 with the corresponding input sample as control, and peaks were annotated by intersection with gene architecture using R package ANNOVAR. HOMER (http://homer.ucsd.edu/homer/motif) was used for de novo followed by localization of the motif with respect to peak summit. StringTie was used to perform expression level for all transcripts and genes from input libraries by calculating FPKM. The differentially expressed transcripts and genes (greater than 1.5-fold, *P* < 0.05) were selected by the R package. m^6^A peaks were visualized using Integrative Genomics Viewer software.

Differentially expressed genes (DEGs) from RNA-seq data and m^6^A hyper-methylated genes from MeRIP-seq data were subjected to functional annotations. Gene Ontology (GO) analysis was performed using PANTHER (https://geneontology.org/) based on three categories: biological process (BP), cellular component (CC), and molecular function (MF). Hallmark gene sets of mice were extracted from R package *msigdbr* (version 7.5.1), and GSEA (Gene Set Enrichment Analysis) analysis was performed by *enricher* function from ClusterProfiler package. The geneset is available at Molecular Signatures Database (MSigDB, https://www.gseamsigdb.org/gsea/downloads.jsp) with either the immunologic signature gene sets (C7) or Hallmark Gene Sets. Enrichment results were visualized using the *barplot* function for the ggplot2 package (version 3.4.4).

### Statistical analysis

Data were presented as means ± SD with *n* per group and a number of experimental replicates indicated in the respective figure legends. The sample size was not calculated using a statistical approach but instead was determined based on previous experimental observation. Animals were randomly assigned to different experimental groups, and no blinding procedures were applied during the animal studies. The variability between the groups under statistical comparisons is comparable. Two-tailed Student’s *t*-test was used for all statistical calculations using GraphPad Prism 9 software. For comparison among different groups, one-way or two-way ANOVA was used. All bar graphs include means with error bars to show the distribution of the data. The level of significance is indicated as *P < 0.05, **P < 0.01, and ***P < 0.001.

## Supplementary information

Supplementary Information

Supplementary Table

Supplementary Data1

Supplementary Data2

Supplementary Data5

## Data Availability

The RNA-seq data and MeRIP-seq generated in this study have been deposited in the Gene Expression Omnibus (GEO) archive under accession GSE270717.
