# Supplementary material for: FTO controls CD8+ T cell survival and effector response by modulating m6A methylation of Fas
Source: Cell Death Dis. 2025 Apr 15;16(1):301. doi: 10.1038/s41419-025-07606-z (PMC12000336; doi:10.1038/s41419-025-07606-z)
Supplement: Supplementary file 1 — Supplementary Information [file 41419_2025_7606_MOESM1_ESM.pdf]

Supplementary Materials for

FTO controls CD8$^+$ T cell survival and effector response by modulating m^{6}A methylation of Fas

Lina Sun[#], Tianzhe Zhang[#], Yao Ge[#], Zhihong Yao[#], Yanhong Su[#], Qianhao Wang, Chen Yang, Boxiao He, Renyi Ding, Cangang Zhang, Linbo Lan, Ruonan Liu, Huanxin Ping, Dan Zhang, Lin Shi, Xiaobo Zhou, Xiaoxuan Jia, Chenming Sun*, Lingli Liang*, Lianjun Zhang* and Baojun Zhang*

Correspondence to: Chenming Sun, cm.sun@xjtu.edu.cn, Lingli Liang, ll2017@xjtu.edu.cn, Lianjun Zhang, zlj@ism.cams.cn and Baojun Zhang, bj.zhang@mail.xjtu.edu.cn

This PDF file includes:

Figures. S1 to S7

Supplementary figures

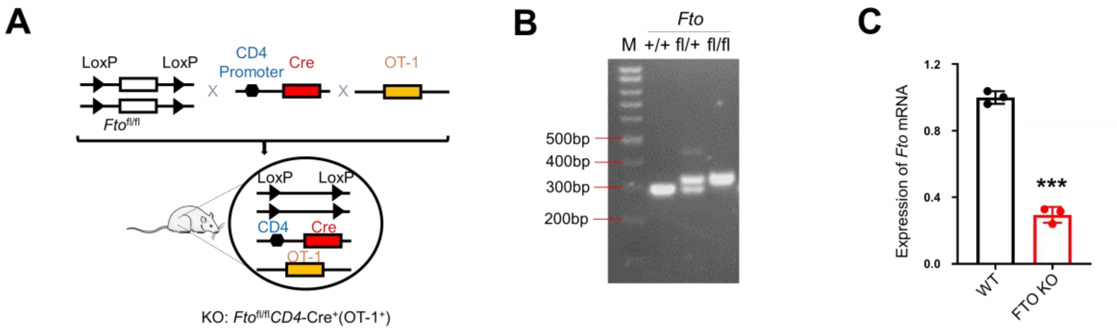

Figure. S1.

Generation of T cell-specific FTO conditional knockout mice. (**A**) Scheme of strategy for generating FTO KO mice. *Fto* flox mice were crossed with CD4-Cre transgenic strain to generate T cell-specific *Fto* knock-out mice (*Fto*$^{fl/fl}$CD4-*Cre*$^{+}$), which were further crossed with OT-1 transgenic mice to produce *Fto*$^{fl/fl}$CD4-Cre^{+}OT-1^{+} mice. (**B**) Genotyping of WT, *Fto*$^{fl/+}$ and *Fto*$^{fl/fl}$ mice by agarose gel electrophoresis of PCR. (**C**) The mRNA level of FTO in CD8^{+} T cells from WT (*Fto*$^{fl/fl}$CD4-*Cre*$^{-}$) and KO (*Fto*$^{fl/fl}$CD4-*Cre*$^{+}$) mice measured by qPCR (n=3). Data are representative of two or three independent experiments shown as the mean ± SD. Statistical testing is depicted as two-sided, unpaired t-tests; ***$P \leq 0.001$.

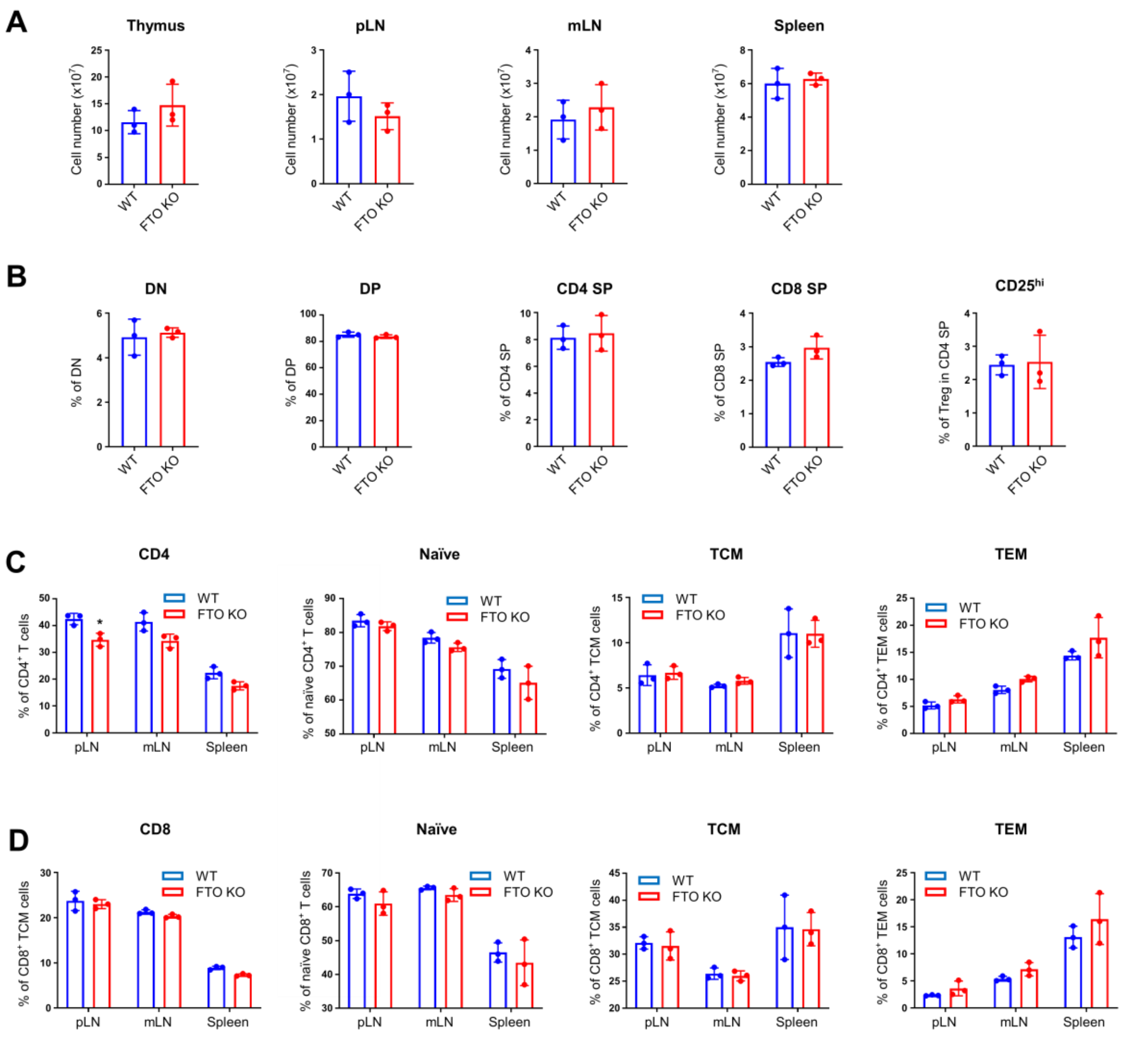

Figure. S2.

FTO deficiency does not affect T cell development in central and peripheral lymphoid tissues.

(**A**) The total cell number in the thymus, peripheral lymph nodes (pLN), mesenteric LN (mLN) and spleen from FTO WT (*Fto*$^{fl/fl}$CD4-*Cre*$^{-}$) and KO (*Fto*$^{fl/fl}$CD4-*Cre*$^{+}$) mice (n=3). (**B**) The percentages (%) of CD4^{-}CD8^{-} double negative (DN), CD4^{+}CD8^{+} double positive (DP), CD4^{+}CD8^{-} single positive (CD4 SP) and CD4^{-}CD8^{+} single positive (CD8 SP) in thymus, and % of CD25^{+} (Treg) cells in CD4 SP cells in WT and KO mice. (**C**) The percentages of total CD4^{+} T cells, naïve (CD62L^{+}CD44^{-}), TCM (CD62L^{+}CD44^{+}) and TEM (CD62L^{-}CD44^{+}) CD4^{+} T cells in the pLN, mLN and spleen in WT and KO mice. (**D**) The percentages of total CD8^{+} T cells, naïve

($CD62L^+CD44^-$), TCM ($CD62L^+CD44^+$) and TEM ($CD62L^-CD44^+$) $CD8^+$ T cells in the pLN, mLN and spleen in WT and KO mice. Data are representative of two or three independent experiments shown as the mean ± SD.

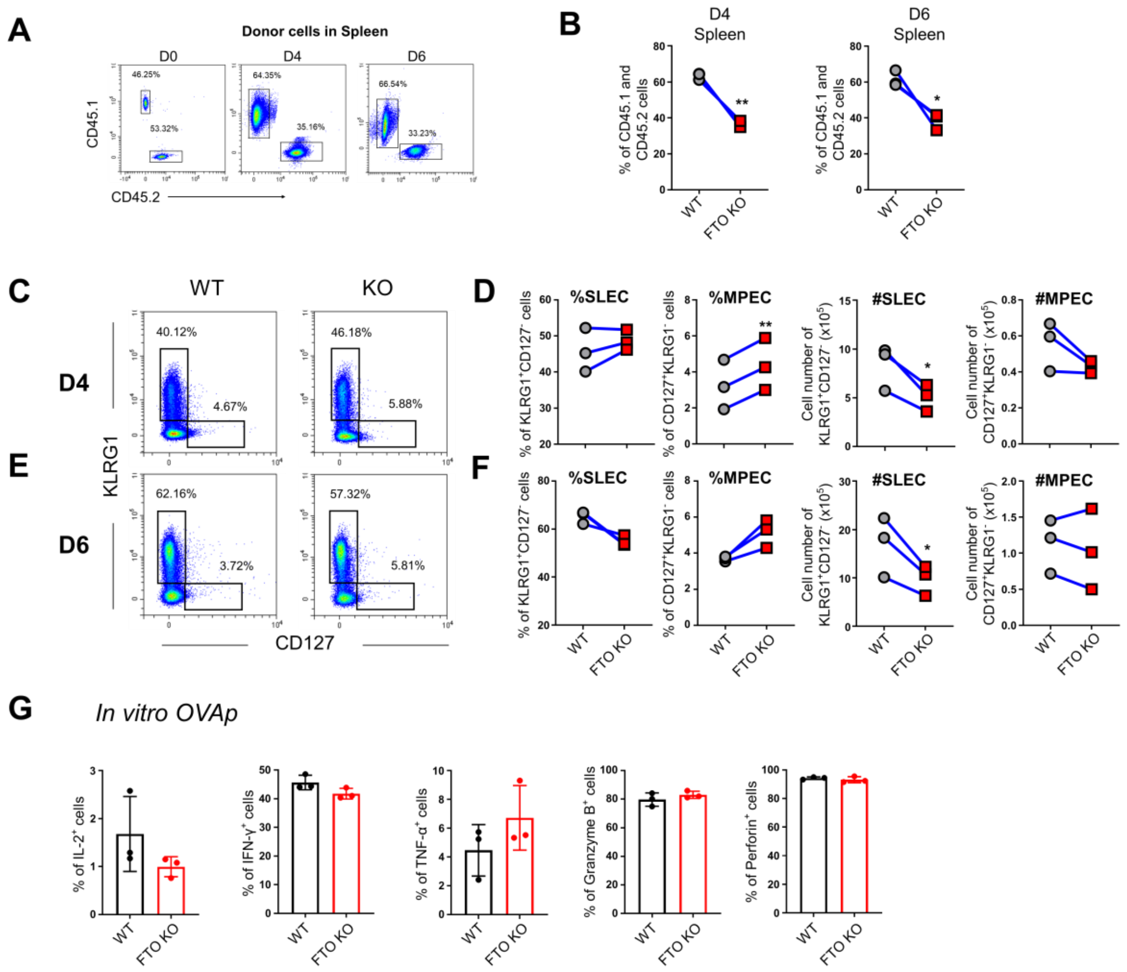

Figure. S3.

FTO deficiency has limited effects on CD8$^+$ T cell differentiation. (**A**) Flow cytometry analysis of splenic CD8$^+$ T cells from WT (*Fto*$^{fl/fl}$CD4-Cre$^-$OT-1$^+$, CD45.1$^+$) and FTO KO (*Fto*$^{fl/fl}$CD4-Cre$^+$OT-1$^+$, CD45.2$^+$) donors before, 4 and 6 days after *LM*-OVA infection in the *in vivo* co-transfer model. (**B**) The proportions of donor-derived CD8$^+$ T cells from WT (CD45.1$^+$) and KO (CD45.2$^+$) groups 4 and 6 days after infection in the spleen from (**A**) (n=3). (**C-F**) Representative flow cytometry plots of KLRG1 and CD127 expression in splenic CD8$^+$ T cells from WT (*Fto*$^{fl/fl}$CD4-Cre$^-$OT-1$^+$, CD45.1$^+$) and FTO KO (*Fto*$^{fl/fl}$CD4-Cre$^+$OT-1$^+$, CD45.2$^+$) donors analyzed 4 (**C**) and 6 (**E**) days post-infection. The proportion (%) and cell number (#) of KLRG1$^+$CD127$^-$ SLECs and KLRG1$^-$CD127$^+$ MPECs in splenic CD8$^+$ T cells from WT (CD45.1$^+$)

and KO ($CD45.2^+$) donors analyzed 4 (**D**) and 6 (**F**) days post-infection (n=3). (**G**) The percentages of $CD8^+$ T cells producing cytokines IL-2, IFN-γ, TNF-α, granzyme B and perforin in $CD8^+$ T cells from WT (*Fto*$^{fl/fl}$CD4-Cre$^-$OT-1$^+$) and FTO KO (*Fto*$^{fl/fl}$CD4-Cre$^+$OT-1$^+$) mice stimulated *in vitro* with $OVA_{257\text{-}264}$ peptide for 24hr. Brefeldin A and monensin were added 4-6hr before cell collection (n=3). Data are representative of two or three independent experiments shown as the mean ± SD. Statistical testing is depicted as two-sided, unpaired t-tests; $^*P \leq 0.05$, $^{**}P \leq 0.01$.

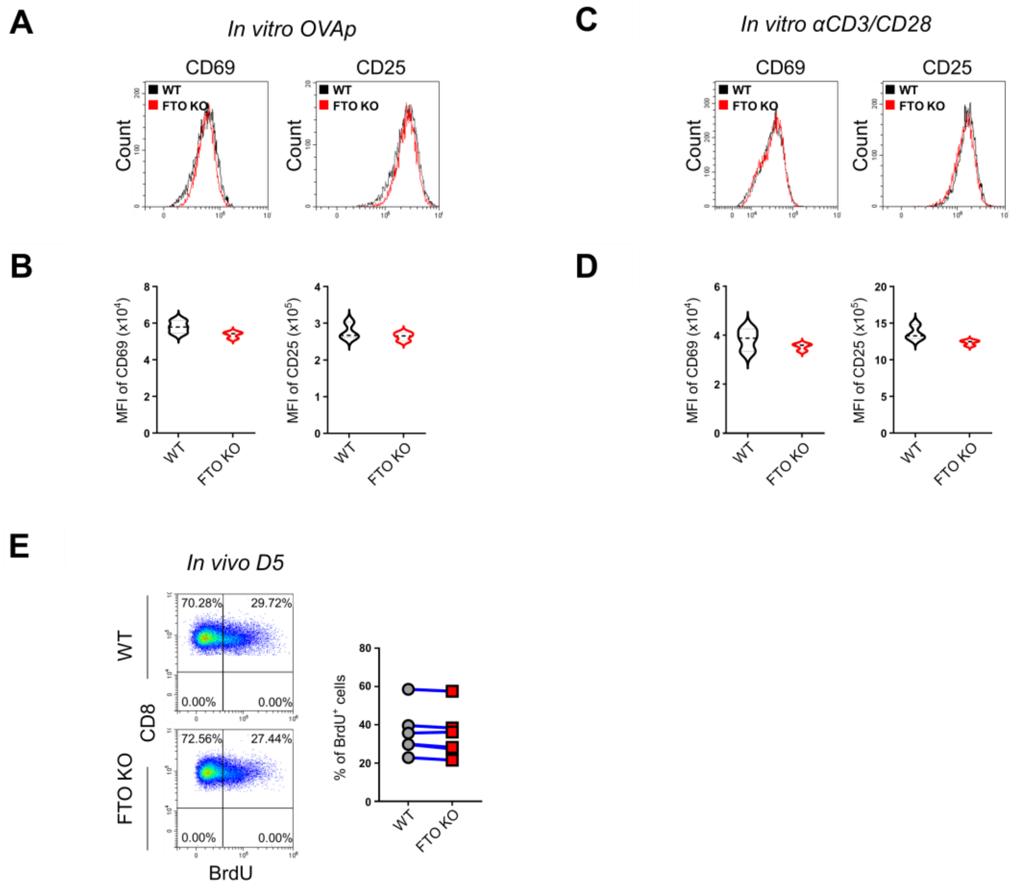

Figure. S4.

FTO deficient $CD8^+$ T cells have comparable cell activation. (**A-B**) Splenocytes from WT (*Fto*$^{fl/fl}$CD4-Cre$^-$OT-1$^+$) and FTO KO (*Fto*$^{fl/fl}$CD4-Cre$^+$OT-1$^+$) mice were isolated and stimulated *in vitro* with $OVA_{257-264}$ peptide for 24hr. Flow cytometry analysis of T cell activation markers CD69 and CD25 expression on WT and KO $CD8^+$ T cells (n=3). (**C-D**) Splenocytes from WT (*Fto*$^{fl/fl}$CD4-Cre$^-$) and FTO KO (*Fto*$^{fl/fl}$CD4-Cre$^+$) mice were isolated and stimulated *in vitro* with anti-CD3/CD28 antibodies for 24hr. Flow cytometry analysis of CD69 and CD25 expression on WT and KO $CD8^+$ T cells (n=3). (**E**) Representative flow cytometry analysis of BrdU expression in WT and FTO KO $CD8^+$ T cells in the *in vivo* co-transfer model (n=5). Data are representative of two or three independent experiments shown as the mean ± SD.

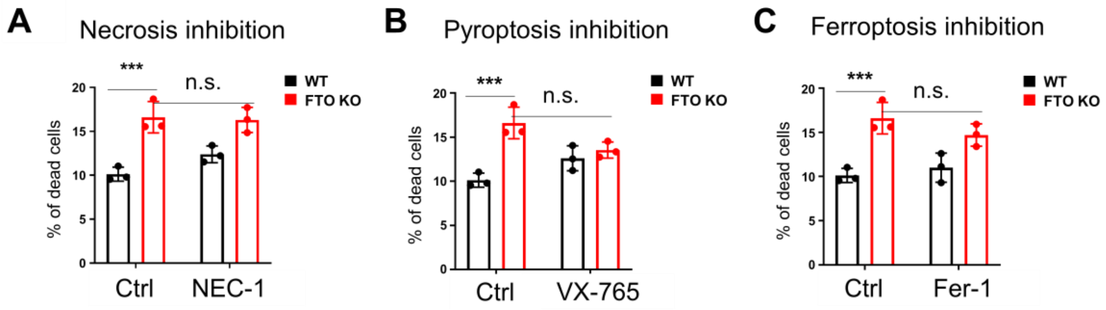

Figure. S5.

FTO deficiency leads to enhanced cell death. Splenocytes from WT (*Fto*$^{fl/fl}$CD4-Cre^{-}) and FTO KO (*Fto*$^{fl/fl}$CD4-Cre^{+}) mice were stimulated with anti-CD3/CD28 antibodies for 24hr in the presence of necrosis inhibitor NEC-1 (**A**), pyroptosis inhibitor VX-765 (**B**) and ferroptosis inhibitor Fer-1 (**C**) before measuring the cell death. Percentages of total dead cells were shown (n=3). n.s. means not significant. Data are representative of two or three independent experiments shown as the mean ± SD. Statistical testing is depicted as two-sided, unpaired t-tests; ***P ≤ 0.001.

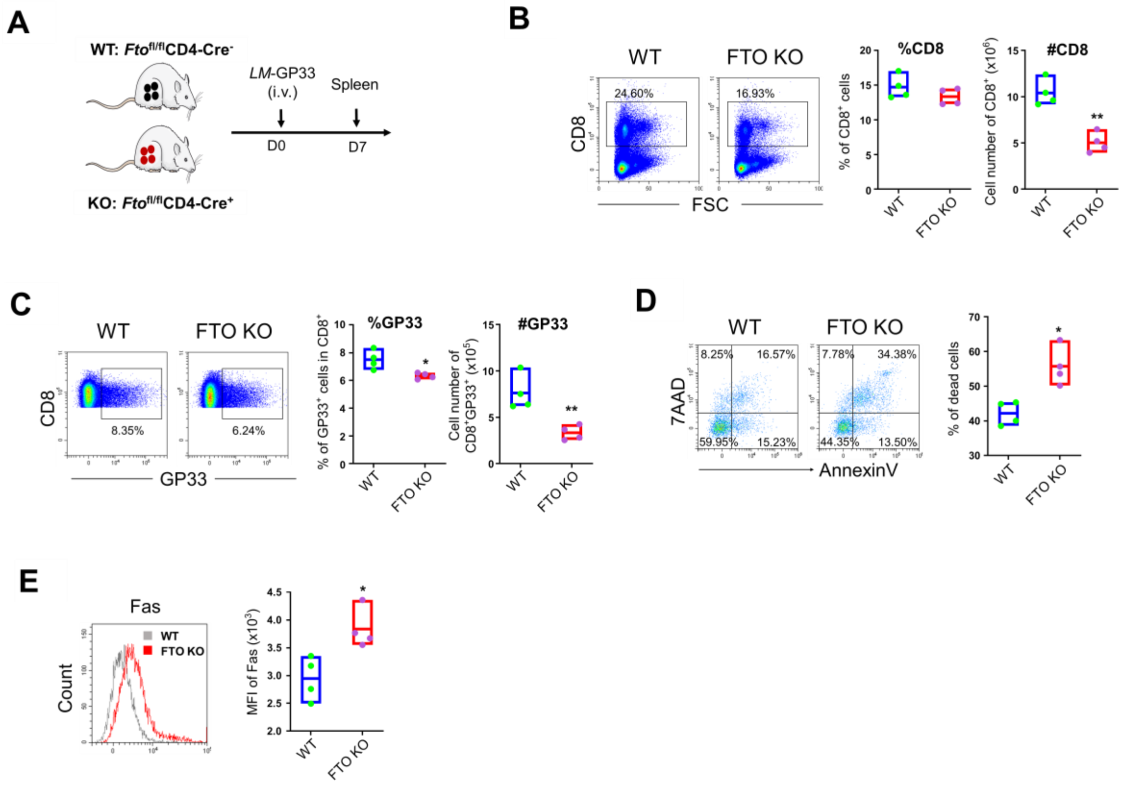

Figure. S6.

FTO affects endogenous CD8^{+} T cell apoptosis. (**A**) Experimental schematic of the direct infection model. WT (*Fto*$^{fl/fl}$CD4-Cre^{-}) and FTO KO (*Fto*$^{fl/fl}$CD4-Cre^{+}) mice were infected by intravenous injection (i.v.) of *LM*-GP33 and CD8^{+} T cells were analyzed in the spleens 7 days post-infection (n=4). (**B**) Flow cytometry analysis of the proportion (%) and cell number (#) of total CD8^{+} T cells from WT and FTO KO mice in (**A**). (**C**) Flow cytometry analysis of the proportion (%) and cell number (#) of antigen-specific tetramer-GP33^{+} CD8^{+} T cells from WT and FTO KO mice in (**A**). (**D**) Flow cytometry analysis of Annexin V and 7AAD expression in CD8^{+} T cells from WT and FTO KO mice in (**A**). (**E**) Flow cytometry analysis of Fas expression in CD8^{+} T cells from WT and FTO KO mice in (**A**). Data are representative of two or three independent experiments shown as the mean ± SD. Statistical testing is depicted as two-sided, unpaired t-tests; *P ≤ 0.05, **P ≤ 0.01.

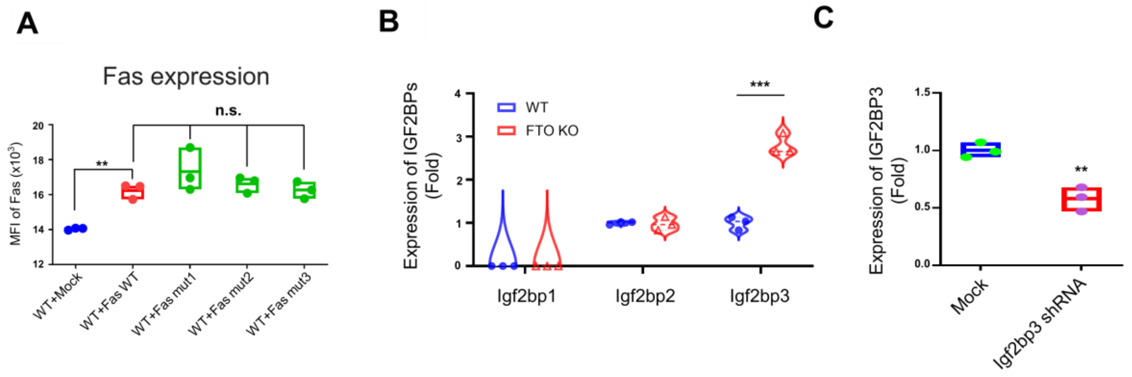

Figure. S7.

FTO regulates Fas expression dependent on IGF2BP3. (**A**) Fas expression levels of WT CD8^{+} T cells overexpressed (OE) with either Mock, *Fas* WT and *Fas* mutant plasmids after stimulation with anti-CD3/CD28 antibodies for 24hr (n=3). n.s. means not significant. (**B**) The mRNA levels of *Igf2bp1*, *Igf2bp2* and *Igf2bp3* were measured by qPCR in WT (*Fto*$^{fl/fl}$CD4-Cre^{-}OT-1^{+}) and FTO KO (*Fto*$^{fl/fl}$CD4-Cre^{+}OT-1^{+}) CD8^{+} T cells isolated from recipient mice 5 days after *LM*-OVA infection in the *in vivo* co-transfer model (n=3). (**C**) The mRNA levels of IGF2BP3 in CD8^{+} T cells transfected with either Mock or *Igf2bp3* shRNA were measured by qPCR (n=3). Data are representative of two or three independent experiments shown as the mean ± SD. Statistical testing is depicted as two-sided, unpaired t-tests or one-way ANOVA; $^{**}P \leq 0.01$, $^{***}P \leq 0.001$.
