# Supplementary material for: FTO controls CD8+ T cell survival and effector response by modulating m6A methylation of Fas
Source: Cell Death Dis. 2025 Apr 15;16(1):301. doi: 10.1038/s41419-025-07606-z (PMC12000336; doi:10.1038/s41419-025-07606-z)
Supplement: Supplementary file 2 — Supplementary Table [file 41419_2025_7606_MOESM2_ESM.docx]

**Supplementary Table 1.** **The binding relationship between IGF2BP3 and Fas predicted by RM2Target website.**

| **RM2Target ID** | **WERs name** | **WERs type** | **Target gene** | **Modification** | **Organism** | **Cell line/Tissue** | **Evidence types/Method** |
| --- | --- | --- | --- | --- | --- | --- | --- |
| RM2Target_991436 | IGF2BP3 | Reader | Fas | m6A | Homo sapiens | REH/Acute myelogenous leukemia (AML) cell line | Protein-RNA binding/iCLIP-seq |
| RM2Target_991438 | IGF2BP3 | Reader | Fas | m6A | Homo sapiens | RS4;11/AML cell line | Protein-RNA binding/iCLIP-seq |
| RM2Target_1682692 | Igf2bp3 | Reader | Fas | m6A | Mus musculus | CD11b^+^ cells/ Splenic AML tumor | Protein-RNA binding/eCLIP-seq |
| RM2Target_991437 | IGF2BP3 | Reader | Fas | m6A | Homo sapiens | PANC-1/ Pancreatic cancer cell line | Expression perturbation/ RNA-seq |
| RM2Target_991439 | IGF2BP3 | Reader | Fas | m6A | Homo sapiens | PL45/Pancreatic cancer cell line | Alternative splicing/RNA-seq |
