# Supplementary figures and images for: FTO controls CD8+ T cell survival and effector response by modulating m6A methylation of Fas
Source: Cell Death Dis. 2025 Apr 15;16(1):301. doi: 10.1038/s41419-025-07606-z (PMC12000336; doi:10.1038/s41419-025-07606-z)

Fig. 3G

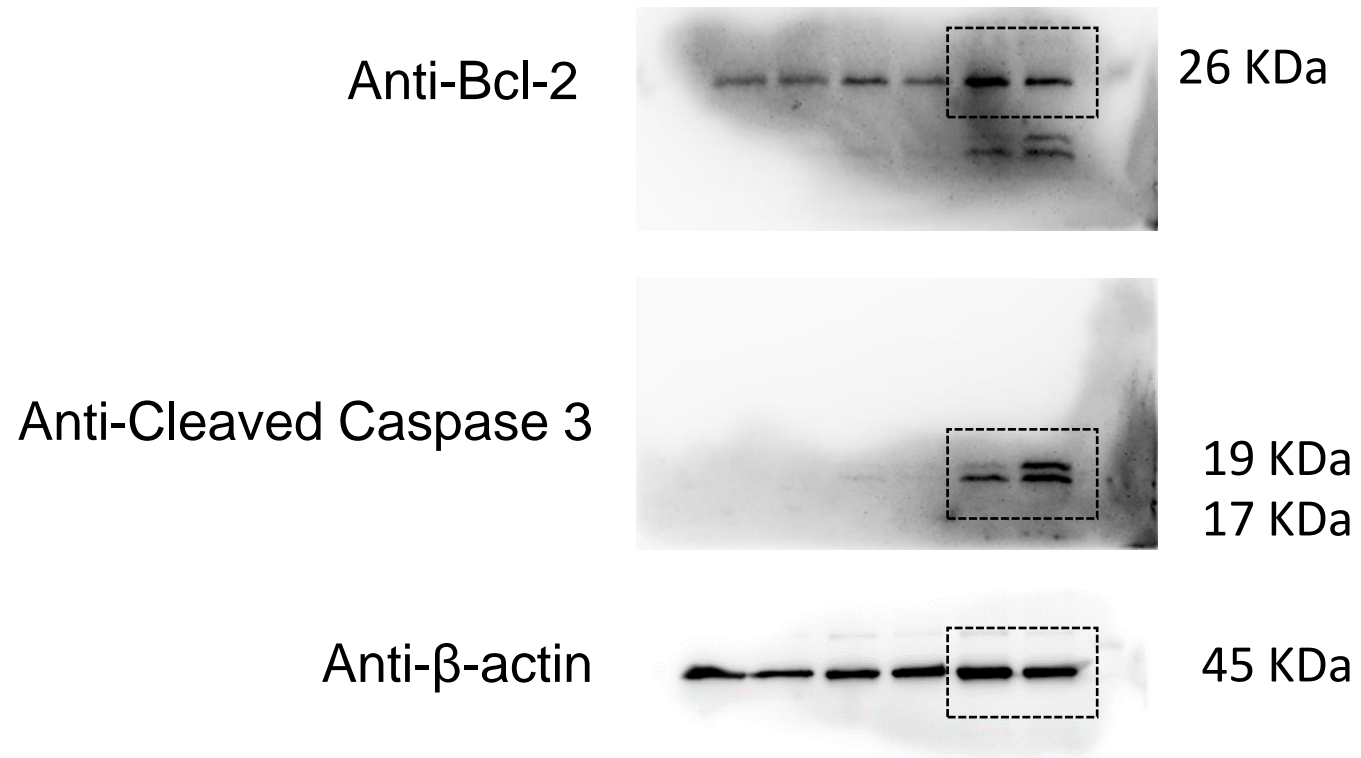

Supplement: Supplementary file 5 — Supplementary Data5 [file 41419_2025_7606_MOESM5_ESM.pdf]
